# Ultrasound-Assisted Deep Eutectic Solvent Extraction of Polysaccharides from *Baeckea frutescens* L.: Hybrid RSM and GA–BP Optimization, Structural Characterization, and Anti-Complementary Activity

**DOI:** 10.3390/molecules31214008

**Published:** 2026-11-06

**Authors:** Ming Bu, Haijun Wang, Yukun Ma, Tianci Xu, Lu Wang, Lina Guo, Jiaming Wang

**Affiliations:** 1 College of Pharmacy, Qiqihar Medical University, Qiqihar 161006, China; 2 Research Institute of Medicine & Pharmacy, Qiqihar Medical University, Qiqihar 161006, China

**Keywords:** *Baeckea frutescens* L., polysaccharides, deep eutectic solvents, extraction process optimization, anti-complementary activity

## Abstract

*Baeckea frutescens* L., a traditional medicinal shrub, is widely applied for the treatment of rheumatism, inflammation and skin diseases. Nevertheless, studies focusing on its therapeutic polysaccharides remain limited. In this work, an ultrasound-assisted deep eutectic solvent (UAE–DES) method was established to extract *B. frutescens* polysaccharides (BFPs). Integrated with single-factor experiments, RSM and GA–BP neural network modeling, the optimal conditions were determined as 35% water content, 52 °C, 31 min, and a liquid–solid ratio of 32:1 mL/g. The maximum BFP yield reached 10.58 ± 0.21%, which was 4.4-fold higher than that achieved with conventional hot water extraction. The choline chloride-urea DES maintained over 83% extraction capacity after five cycles, demonstrating favorable reusability. Kinetic analysis based on Fick’s second law indicated diffusion-controlled extraction with low activation energy. UAE–DES–BFPs exhibited reduced molecular weight, altered monosaccharide composition and improved thermal stability. Correspondingly, UAE–DES–BFPs showed superior anti-complementary activity with CH_50_ and AP_50_ values of 120.13 μg/mL and 255.54 μg/mL. Both polysaccharides suppressed complement activation via C1q, C3, C4, C5 and C9, and UAE–DES–BFPs more effectively inhibited C3/C4 deposition. This study confirms that UAE–DES is an efficient strategy for bioactive BFP preparation, supporting the high-value utilization of *B. frutescens*.

## 1. Introduction

*Baeckea frutescens* L. is a medicinal shrub widely distributed in southeastern China, India, and Australia, with well-documented medicinal and economic value [1]. As a traditional herbal remedy, it is clinically applied to treat rheumatism, inflammatory disorders, fever, and skin diseases [2]. Current phytochemical investigations of *B. frutescens* have predominantly focused on small-molecule metabolites, including essential oils, flavonoids, and phloroglucinols [3,4]. In contrast, polysaccharide-related research remains insufficient, despite the versatile bioactivities of plant polysaccharides such as immunomodulation, antioxidation, and anti-inflammation activities [5]. Accordingly, exploring the extraction, structural characteristics, and bioactivity of *B. frutescens* polysaccharides (BFPs) is essential to facilitate the comprehensive utilization of this medicinal plant.

Extraction techniques largely determine the yield, structural integrity, and biological performance of plant polysaccharides [6]. Conventional hot water extraction (HWE) is widely adopted due to its low cost and simple operation, yet it is inefficient, requires a long time, and has the potential to thermally degrade thermosensitive polysaccharides. Enzyme-assisted and microwave-assisted extraction methods can improve extraction performance to a certain extent, but their applications are limited by high enzyme costs, strict operational requirements, or uneven heating [7,8,9,10]. As an alternative, ultrasound-assisted extraction (UAE) disrupts plant cell walls via acoustic cavitation, accelerating mass transfer and shortening extraction time under mild conditions, thus emerging as a reliable technique for polysaccharide preparation [11]. Nevertheless, conventional UAE relies on water or volatile organic solvents, which restrict its extraction efficiency and sustainability.

Deep eutectic solvents (DESs) have recently emerged as promising green extraction media owing to their low toxicity, negligible volatility, simple preparation, and tunable solvation capacity [12]. The combination of UAE and DESs achieves synergistic effects, integrating the cavitation disruption of ultrasound and the strong solubilizing ability of DESs, thereby effectively improving the extraction efficiency of plant bioactive components [13]. Recent studies have verified the superior performance of UAE–DES in polysaccharide extraction. For example, UAE–DES extraction of *Gymnema sylvestre* polysaccharides achieved a 4.4-fold higher yield than conventional water extraction, demonstrating great potential for polysaccharide enrichment [7]. Therefore, the UAE–DES strategy provides an efficient and sustainable alternative for green extraction of natural polysaccharides.

Process optimization and kinetic analysis are critical for improving extraction efficiency and revealing mass transfer mechanisms. Response surface methodology (RSM) is commonly used to analyze interactions among extraction variables, while its quadratic polynomial model often fails to accurately describe the highly nonlinear relationships of complex extraction systems [14]. The genetic algorithm–back propagation (GA–BP) neural network possesses superior nonlinear fitting capability, enabling the more precise prediction of optimal extraction conditions [15]. The integration of RSM and GA–BP can compensate for the limitations of single-model optimization. Furthermore, kinetic analysis based on Fick’s second law helps elucidate the diffusion and mass transfer mechanisms during polysaccharide release [16]. In this study, UAE–DES was employed to extract BFPs, and the extraction parameters were systematically optimized via a combined RSM and GA–BP approach. The extraction kinetics were modeled to calculate kinetic constants and apparent activation energy. The structural, compositional, and thermal properties of UAE–DES–BFPs were characterized and compared with those of traditional HWE–BFPs. Scanning electron microscopy was used to observe the morphological changes in the plant residues after different extraction treatments. The anti-complementary activities of BFPs were evaluated via classical and alternative complement pathways, and the underlying inhibitory mechanism was further explored through complement-depletion assays and ELISA-based C3/C4 deposition quantification. Moreover, the recyclability of DES was assessed to verify the sustainability of the extraction system. This study aims to provide a feasible theoretical basis for the efficient green extraction and high-value utilization of B. frutescens polysaccharides.

## 2. Results and Discussion

### 2.1. Screening and Selection of DESs

**Figure 1:**
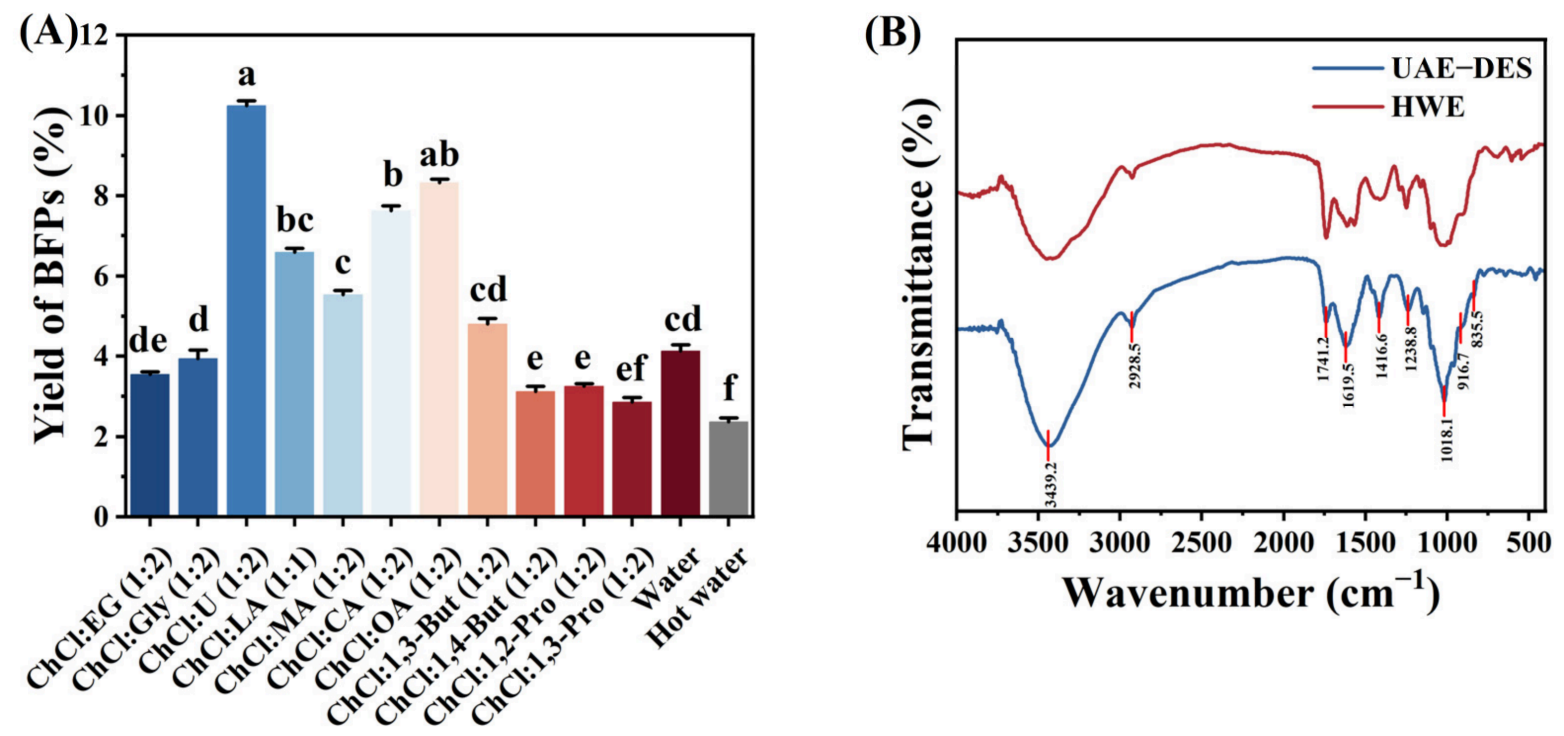
DES screening and FT-IR analysis of BFPs. (**A**) DES screening for BFP yield (n = 3). Abbreviations: ChCl, choline chloride; EG, ethylene glycol; Gly, glycerol; U, urea; LA, lactic acid; MA, malic acid; CA, citric acid; OA, oxalic acid; 1,3-But, 1,3-butanediol; 1,4-But, 1,4-butanediol; 1,2-Pro, 1,2-propanediol; 1,3-Pro, 1,3-propanediol. (**B**) FT-IR spectra of BFPs obtained under different extraction conditions.

Choline chloride was chosen as the hydrogen-bond acceptor due to its low toxicity, good biodegradability, and extensive application within natural product extraction [17]. Among the eleven DES formulations screened under identical conditions, the choline chloride–urea system (ChCl–U) afforded the highest BFP yield (10.24 ± 0.12%, Figure 1A) and was therefore used for all subsequent experiments. The observed performance advantage arises from the strong hydrogen-bond interactions established between choline chloride and urea. These interactions help disintegrate plant cell-wall structures and boost the dissolution of target polysaccharides [18].

Residual DES components were considered effectively removed by 48 h dialysis against distilled water (3500 Da cutoff), since both choline chloride (139.6 Da) and urea (60 Da) lie well below the membrane molecular weight cutoff. The purified BFPs displayed no characteristic FT-IR absorption bands attributable to free urea or choline chloride, providing spectroscopic confirmation of adequate DES removal (Figure 1B).

To separate the contributions of DES and ultrasound, UAE–W was conducted under identical sonication parameters using distilled water in place of DES. The UAE–W yield reached 4.12 ± 0.15%, which was 74.6% higher than conventional hot water extraction (2.36 ± 0.10%), confirming that ultrasonic cavitation alone provides a measurable enhancement. Nevertheless, UAE–DES (10.24 ± 0.12%) further exceeded UAE–W by 148.5%, demonstrating that DES solvation contributes an additional gain beyond the physical effects of sonication. The 4.3-fold yield advantage of UAE–DES over HWE thus reflects the combined action of DES chemistry and ultrasonic cavitation rather than either factor in isolation.

### 2.2. Single-Factor Experiments

**Figure 2:**
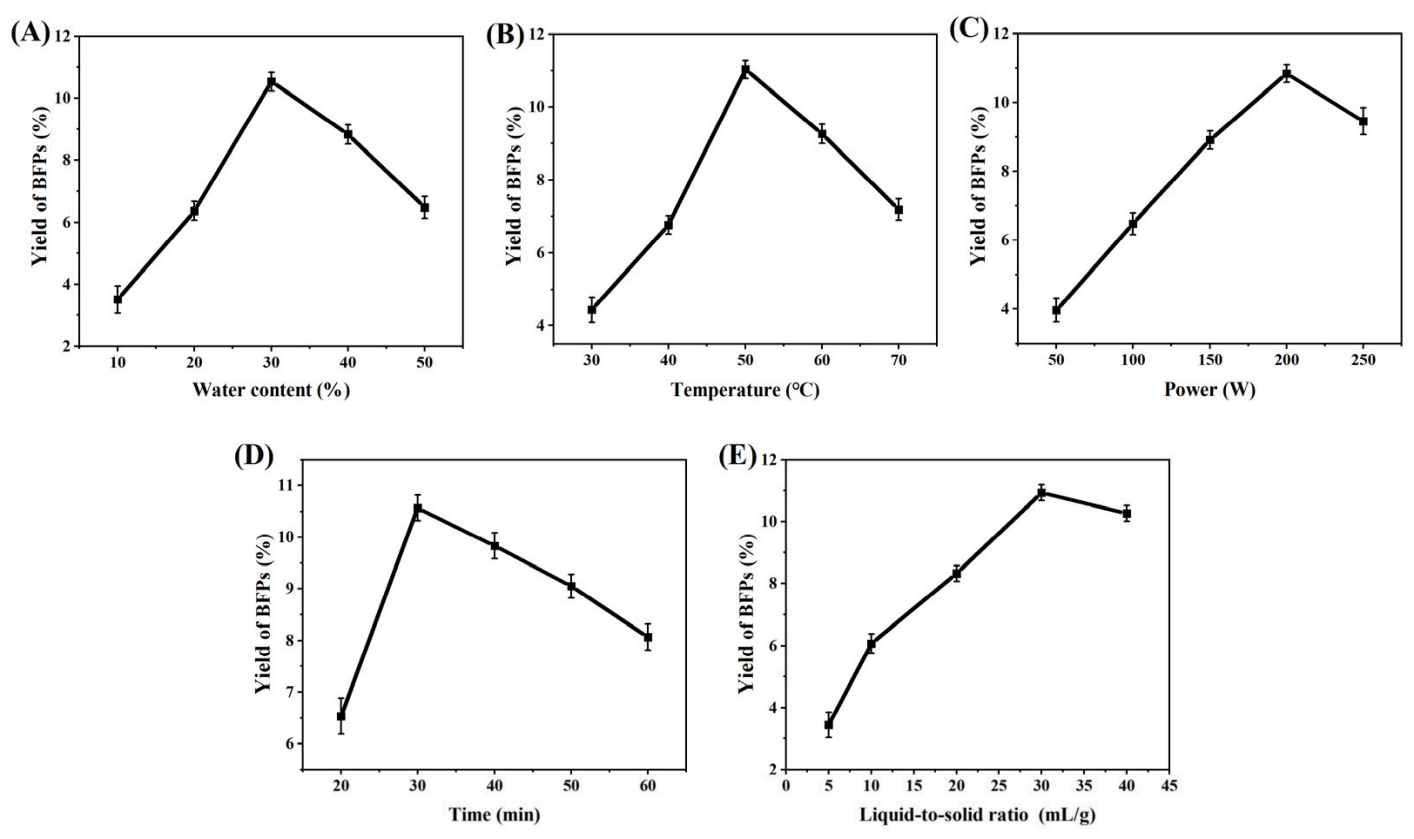
Single-factor experimental results for BFPs extraction (n = 3). (**A**) water content; (**B**) ultrasound temperature; (**C**) ultrasonic power; (**D**) ultrasound time; (**E**) liquid–solid ratio on BFPs yield.

Variations in extraction conditions can impact extraction efficiency. Thus, a single-factor experiment was designed to explore the proper factor levels. The effects of water content, ultrasound temperature, ultrasonic power, ultrasound time, and liquid–solid ratio on BFP yield are presented in Figure 2A–E.

As shown in Figure 2A, water content significantly influenced the BFP extraction yield. The extraction yield obtained at 30% water content (10.53 ± 0.30%) exceeded the value measured at 10% water content (3.50 ± 0.43%). This phenomenon may stem from decreased solvent viscosity, which facilitates mass transfer. Nevertheless, once water content rose above 30%, polysaccharide extraction was inhibited. Excessive dilution broke the hydrogen-bond network formed between choline chloride and urea, reducing the solvation ability of DES [19]. On the basis of this result, 30% water content was chosen as the suitable extraction condition.

Figure 2B illustrates the effect of ultrasound temperature on BFP yield. The yield increased from 4.43 ± 0.31% to 11.03 ± 0.25% as temperature was elevated from 30 °C to 50 °C, before falling to 7.19 ± 0.29% at 70 °C. Higher temperature accelerates molecular motion and reduces solvent viscosity, promoting mass transfer. However, excessive temperature rise is likely to cause degradation of polysaccharide chains and disturb the hydrogen-bond interactions between DES solvent and polysaccharide molecules [20]. For this reason, 50 °C was identified as the most favorable extraction temperature.

Figure 2C illustrates the influence of ultrasonic power on BFP extraction yield. The yield rose from 3.97 ± 0.35% to 10.83 ± 0.24% with the increase in ultrasonic power from 50 W to 200 W. This yield gain can be largely attributed to ultrasonic cavitation. It creates micro-jets and shear stresses capable of disintegrating plant cell-wall structures and improving solvent infiltration within plant matrices [21]. However, excessive power at 250 W caused a slight decrease in yield, which may have been due to free radical generation and localized overheating that could damage the polysaccharide structure. Accordingly, 200 W was determined to be the optimum ultrasonic power for extraction.

Figure 2D depicts the effect of ultrasound time on BFP yield. The extraction yield grew from 6.53 ± 0.33% to 10.57 ± 0.25% as the extraction period was prolonged from 20 min to 30 min; further extension up to 60 min produced no obvious yield improvement. This result indicates that 30 min is sufficient to achieve effective mass transfer and the near-complete release of the polysaccharides from the plant matrix. Beyond this time point, the concentration gradient between solid matrix and extraction solvent approached equilibrium, so prolonging extraction provided no additional benefit. Thus, 30 min was determined to be the optimum sonication time for extraction.

As illustrated in Figure 2E, the liquid–solid ratio exerted a marked impact on the extraction yield of BFPs. Elevating this ratio from 5:1 up to 30:1 mL/g generated yield enhancement from 3.43 ± 0.40% to 10.93 ± 0.23%. Nevertheless, further raising the ratio to 40:1 mL/g triggered a gradual yield reduction to 10.27 ± 0.25%. A higher liquid–solid ratio increases the concentration-gradient driving force for mass transfer and favors polysaccharide liberation. Nevertheless, an excessively high ratio dilutes DES medium and reduces cavitation intensity per unit volume, leading to deteriorated extraction performance. Thus, a liquid–solid ratio of 30:1 mL/g was chosen as the optimum condition.

Based on the single-factor experimental results, the preliminary central extraction parameters for BFPs were identified as water content 30%, ultrasound temperature 50 °C, ultrasonic power 200 W, ultrasound time 30 min, and liquid–solid ratio 30:1 mL/g. These values were used as the center points for subsequent RSM optimization, with four variables, water content, temperature, time, and liquid–solid ratio, selected as independent factors due to their significant influence on extraction yield, while ultrasonic power was fixed at its optimal level of 200 W.

### 2.3. Response Surface Methodology Optimization

Single-factor experimental findings served as the basis for further optimization via a four-factor, three-level Box–Behnken design (BBD). All obtained experimental data are listed in Table 1. Multiple regression analysis was then performed to establish the quadratic polynomial equation for predictive modeling:

Y = +10.95 + 0.1892A + 0.2467B + 0.1392C + 0.1183D + 0.0800AB − 0.0850AC + 0.2275AD + 0.3350BC − 0.0600BD + 0.0275CD − 1.69A^2^ − 1.11B^2^ − 1.12C^2^ − 0.6423D^2^.

As shown in Table 2, the model yielded a *p*-value below 0.001, pointing to high statistical significance, whereas the lack-of-fit term returned a *p*-value of 0.9982 (>0.05), implying non-significant lack-of-fit. These outcomes verify the robustness of the constructed model. An R^2^ of 0.9881 reflects favorable agreement between experimentally measured and model predicted BFP yields. According to F-value magnitudes, the factors influencing extraction yield can be ordered as ultrasound temperature (B) > water content (A) > ultrasound time (C) > liquid–solid ratio (D). The linear terms (A, B, C), quadratic terms (A^2^, B^2^, C^2^, D^2^), and interaction BC attained high significance (*p* < 0.01). By contrast, factor D and the AD interaction exhibited significant effects at *p* < 0.05.

**Figure 3:**
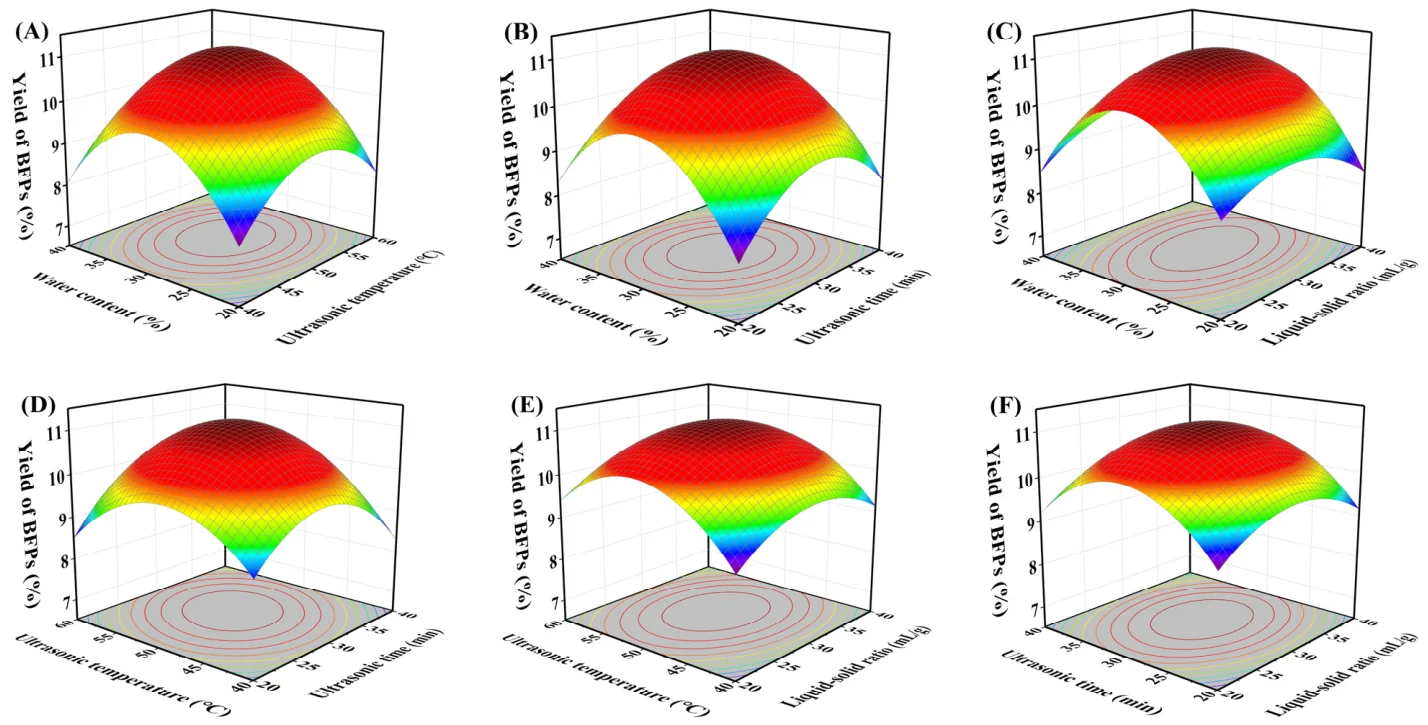
The 3D response surface and contour plots showing interactive effects among variables affecting BFP extraction: (**A**) water content and ultrasonic temperature; (**B**) water content and ultrasonic time; (**C**) water content and liquid–solid ratio; (**D**) ultrasonic temperature and ultrasonic time; (**E**) ultrasonic temperature and liquid–solid ratio; (**F**) ultrasonic time and liquid–solid ratio.

The 3D response surface and contour plots (Figure 3) visualize how each factor and their interactions influenced BFP yield. Water content and liquid–solid ratio showed a strong synergistic interaction (Figure 3C). Increasing water content alone reduced DES viscosity and improved solvent penetration, while a higher liquid–solid ratio provided a greater concentration gradient and more solvent volume. When both factors were low, the solvent remained viscous and insufficient; raising either one alone produced limited improvement, whereas increasing both in tandem produced a synergistic enhancement in effective mass transfer. The interaction between temperature and extraction time was also significant (Figure 3D). Higher temperature lowered DES viscosity and accelerated molecular motion, so the same contact time yielded more polysaccharide; at lower temperature, extending the contact time partially compensated for the slower diffusion. This complementary relationship between thermal and temporal effects explained why the two factors jointly influenced yield more strongly than either alone. In comparison, Figure 3A,B,E,F show nearly circular contours and a relatively gentle slope, consistent with the non-significant ANOVA result for the AB, AC, BD, and CD interactions, respectively.

According to the outputs from model optimization, the optimum operating parameters for BFP extraction were determined as follows: water content 35.88%, ultrasonic temperature 51.37 °C, ultrasonic duration 30.63 min, and liquid–solid ratio 31.91:1 mL/g, corresponding to a predicted extraction yield of 10.52%. Taking experimental operability into account, these theoretical parameters were revised to 36%, 51 °C, 31 min and 32:1 mL/g. Under such adjusted conditions, the experimental BFP yield reached 10.56 ± 0.17%, showing good consistency with the model-predicted value and hence verifying the dependability of the BBD design. Notably, the UAE–DES system provided a 4.4-fold higher polysaccharide yield compared with conventional hot water extraction (2.36%), which demonstrates the prominent superiority of this UAE–DES extraction approach.

### 2.4. GA–BP Neural Network Optimization and Comparison with RSM

**Figure 4:**
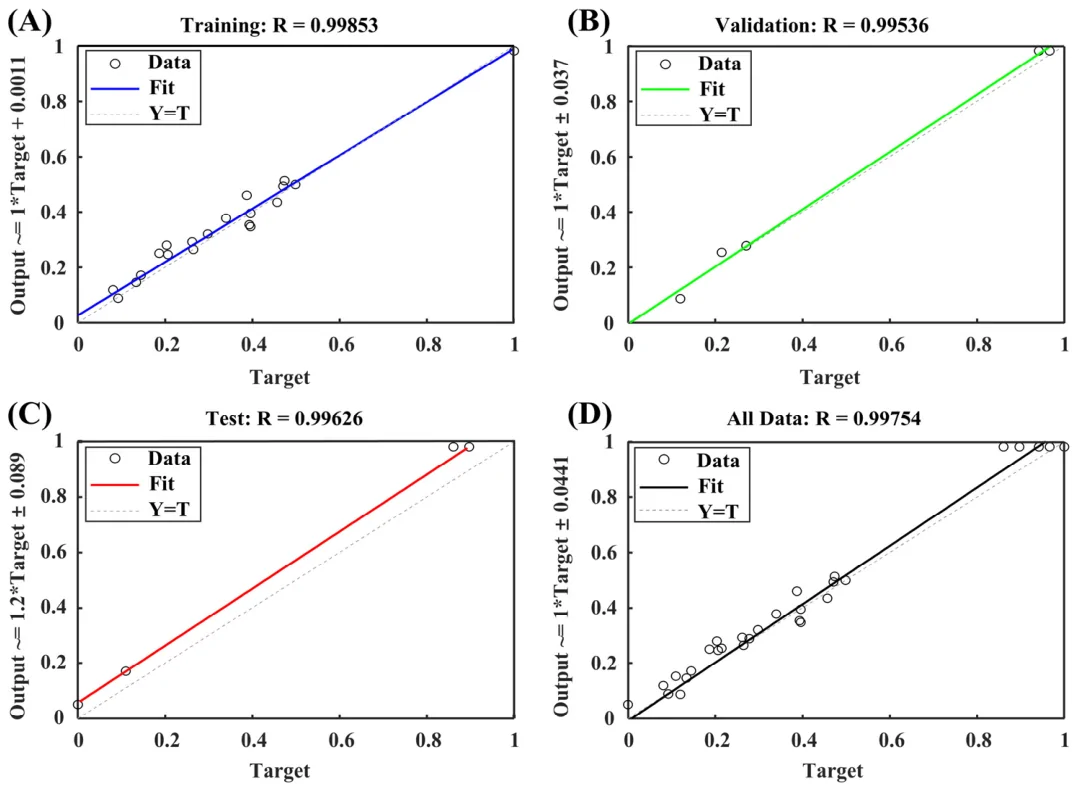
Parity plots of GA–BP-predicted and experimental BFP yields for (**A**) training set, (**B**) validation set, (**C**) test set, and (**D**) all datasets.

The RSM experimental data were used to train the GA–BP neural network to predict BFP yield more intelligently. Figure 4A–D show the parity plots for the training, validation, test, and overall datasets. The correlation coefficients (R) were 0.99853, 0.99536, 0.99626, and 0.99754 for the training, validation, test, and overall sets, respectively. The test set R was 0.99626, indicating good predictive generalization.

**Figure 5:**
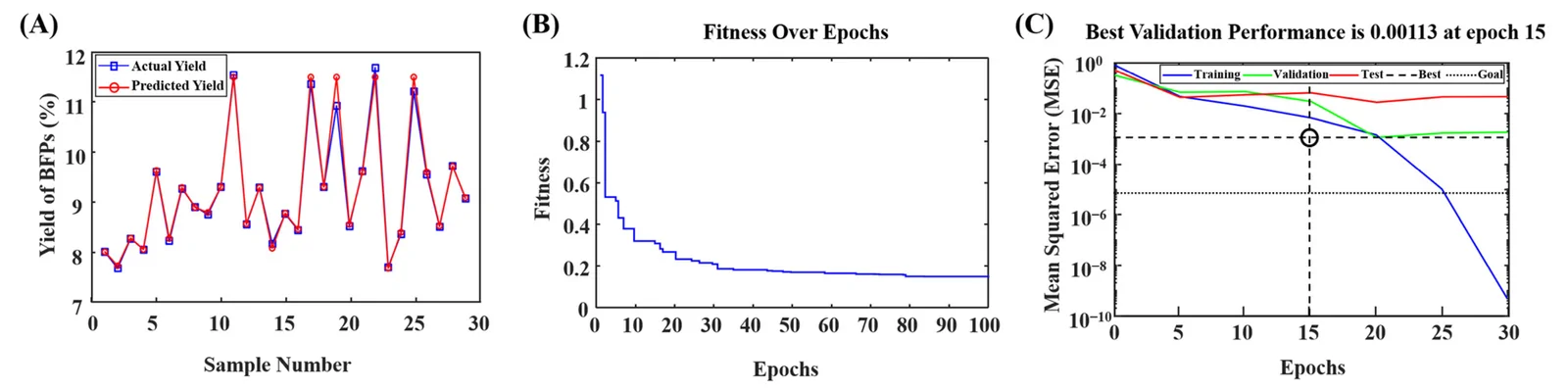
(**A**) Comparison curves between experimental and predicted BFP yields, (**B**) fitness iteration curve of genetic algorithm, and (**C**) MSE descent curve of optimal validation model.

Figure 5A compares the predicted and experimental BFP yields across all 29 runs, showing close agreement throughout. The GA fitness curve (Figure 5B) declined rapidly in the initial generations and then plateaued, indicating efficient convergence toward an optimal set of initial weights and thresholds. The MSE evolution during training (Figure 5C) showed a sharp initial decrease, reaching the lowest validation MSE of 0.00113 at epoch 15. After epoch 15, no rebound was observed for the validation error, and the test error stayed at a low, steady level. These observations verify the network’s generalization ability and the absence of over-fitting behavior.

The GA–BP model was used to predict the optimal extraction conditions. The predicted optimum was 35.46% water content, 51.68 °C, 30.64 min extraction time, and 31.53:1 mL/g liquid–solid ratio, with a predicted yield of 10.55%. For practical validation, these parameters were adjusted to 35% water content, 52 °C, 31 min, and 32:1 mL/g. Under these adjusted conditions, the experimentally determined yield was 10.58 ± 0.21%, showing close agreement with the model prediction.

To make a further comparison of the predictive capacity between the two modeling strategies, leave-one-out cross-validation (LOOCV) was applied across all 29 experimental datasets. Within each fold, both the RSM and GA–BP models were refitted using 28 samples so as to generate predictions for the one single withheld sample. Absolute prediction errors obtained over the 29 folds were subsequently analyzed via the Wilcoxon signed-rank test. As outlined in Table 3, the GA–BP model yielded a higher correlation coefficient (R^2^ = 0.9919 versus 0.9881) as well as smaller prediction errors (RMSE = 0.0722 versus 0.0870; MAE = 0.0428 versus 0.0621) relative to the RSM approach. The Wilcoxon test showed that the GA–BP prediction errors were significantly smaller (*p* = 0.0124), indicating a statistically meaningful improvement in predictive accuracy under the current experimental conditions. Notably, both models predicted nearly identical optimal conditions, and the experimentally validated yields were 10.56 ± 0.17% (RSM) and 10.58 ± 0.21% (GA–BP), a difference within experimental error. This convergence between two independent modeling approaches further supports the reliability of the optimized conditions.

### 2.5. Extraction Kinetics Analysis

#### 2.5.1. Rate Constant

**Figure 6:**
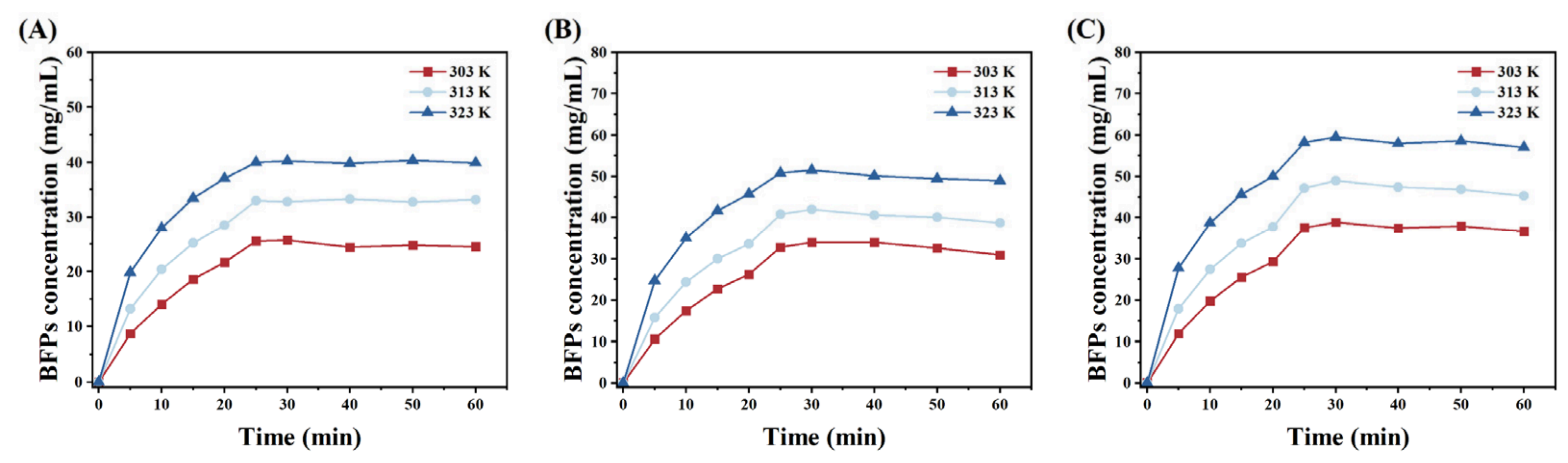
Time courses of polysaccharide concentration (C_t_, mg/mL) extracted at different temperatures and liquid–solid ratios ((**A**): 10 mL/g, (**B**): 20 mL/g, (**C**): 30 mL/g).

**Figure 7:**
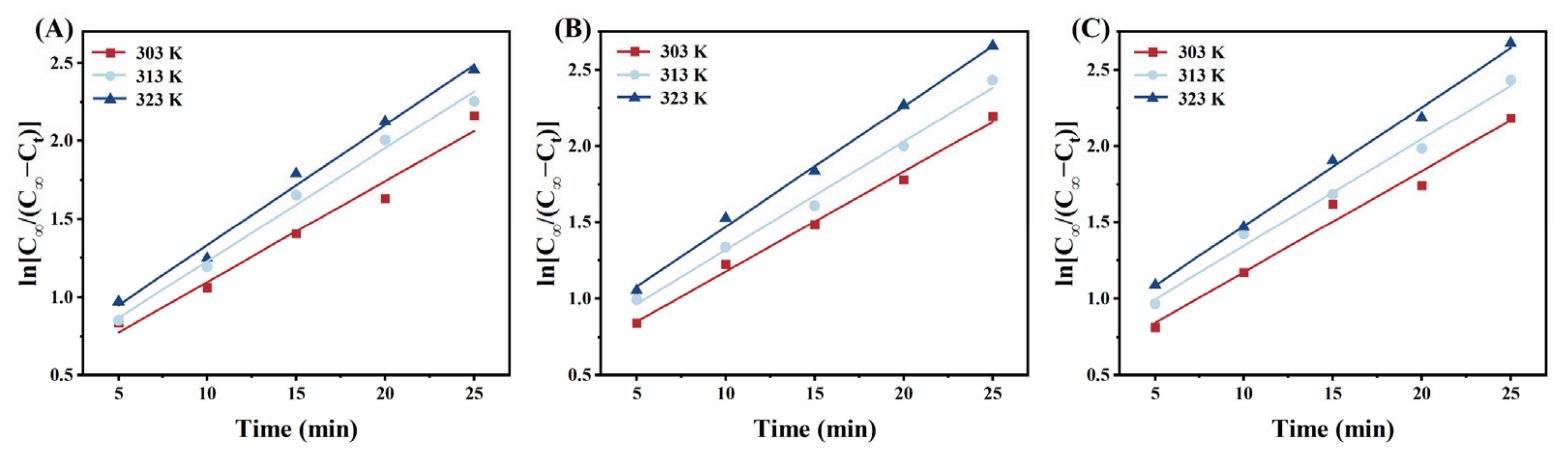
Correlation between ln[C_∞_/(C_∞_−C_t_)] and extraction time at different temperatures and distinct liquid–solid ratios ((**A**): 10 mL/g, (**B**): 20 mL/g, (**C**): 30 mL/g).

The polysaccharide concentration increased progressively during the initial 25 min and then stabilized, with minimal further variation observed over the remaining extraction period. The stabilized concentration was taken as C_∞_ (Figure 6). The unsteady-state spherical diffusion model derived from Fick’s second law was fitted against the experimental kinetic data. Linear regression was performed between ln[C_∞_/(C_∞_ − C_t_)] and extraction time, which exhibited satisfactory fitting quality. All correlation coefficients exceeded 0.97 (Table 4). Obvious differences in fitting slopes under different experimental conditions can be seen from the linear plots presented in Figure 7.

Temperature exerted a consistent positive promoting effect on the extraction rate constant *k*. Elevated temperature reduced DES viscosity, intensified ultrasonic cavitation fragmentation of plant cell walls, and weakened intermolecular interactions between polysaccharide chains and tissue matrices, thereby accelerating the outward diffusion of target polysaccharides [22]. The liquid–solid ratio produced a non-monotonic regulatory effect on k. A moderately increased solvent dosage enlarged solid–liquid concentration gradients to boost mass transfer driving force, yet excessive DES volume absorbed ultrasonic acoustic energy and attenuated cavitation efficiency, partially offsetting the favorable diffusion effect [23]. At a fixed temperature of 323 K, the rate constant k slightly increased from 0.0769 to 0.0787 min^−1^, and then slightly decreased to 0.0778 min^−1^ at 30:1 mL/g. Among all tested combinations, the 20:1 mL/g liquid–solid ratio at 323 K delivered the maximum *k* value.

#### 2.5.2. Relative Residual Rate

**Figure 8:**
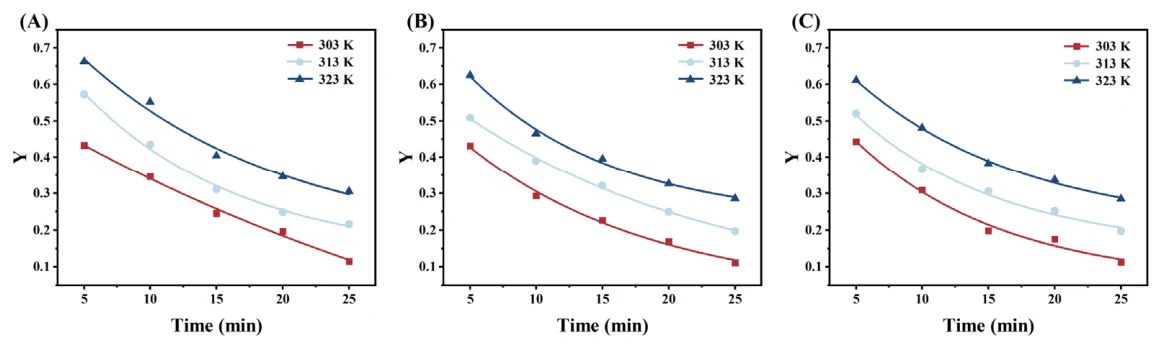
Variations in Y and time for different temperatures and liquid–solid ratios ((**A**): 10 mL/g, (**B**): 20 mL/g, (**C**): 30 mL/g).

To cross-validate the reliability of linear diffusion fitting, the residual fraction model Y = A ⋅ exp^(−*k*t)^ was adopted for nonlinear regression. All exponential fitting results are listed in Table 5. The decay tendency of residual polysaccharide fraction under varied conditions is visually presented in Figure 8.

The decay curves exhibited similar evolutionary characteristics across all groups: a steep drop within the initial 20 min, followed by a gentle plateau in the later extraction phase. The rapid decline in the early stage corresponds to the fast elution of polysaccharides located on particle surfaces and shallow pores [24]. By contrast, the slowed decay after 20 min arose from restricted diffusion of tightly bound polysaccharides embedded within dense cell-wall matrices. Notably, the variation trend in the rate constant *k* obtained from exponential fitting agreed well with the results from linear regression.

#### 2.5.3. Apparent Activation Energy

**Figure 9:**
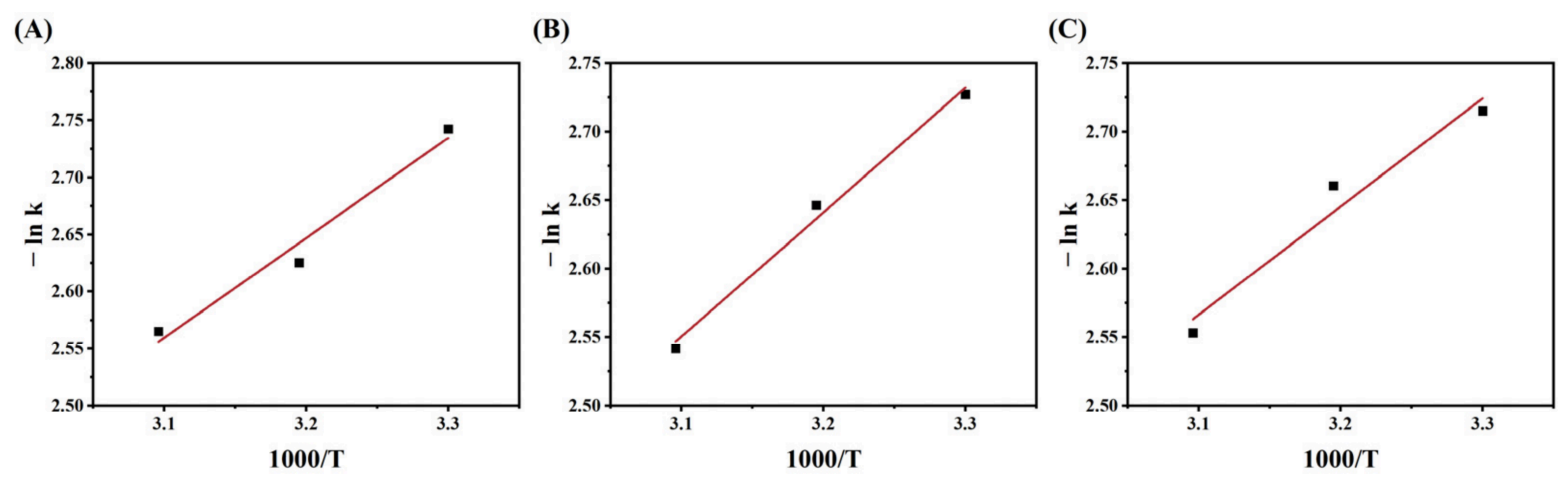
Relationship between −lnk and 1000/T ((**A**): 10 mL/g, (**B**): 20 mL/g, (**C**): 30 mL/g).

The Arrhenius transformation −lnk=E_a_/(RT) − lnA was adopted to quantify the temperature-dependent sensitivity of mass-transfer resistance. Linear regression results for each liquid–solid ratio are summarized in Table 6. Linear fitting plots of −ln *k* against 1000/T are presented in Figure 9.

The calculated apparent activation energy Ea values ranged from 6.56 to 7.61 kJ/mol. According to well-established mass-transfer criteria, extraction processes with Ea < 15 kJ/mol are governed by internal solid–liquid diffusion instead of chemical bond cleavage. This observation suggests an apparent diffusion-controlled extraction behavior for this UAE–DES system [25]. The minimum Ea of 6.56 kJ/mol was obtained at 30:1 mL/g, suggesting that this solvent dosage minimized mass-transfer barriers. By comparison, insufficient DES supply cannot fully solvate intracellular polysaccharides and gives rise to higher diffusion resistance. All Arrhenius fittings exhibited good linearity with R^2^ > 0.96. The literature values for the apparent activation energy for polysaccharide hot water extraction typically fall within 12–25 kJ/mol [26,27]. The considerably lower E_a_ observed in this work further highlights the prominent mass-transfer-enhancing effect originating from the complementary interplay between DES medium and sonication.

### 2.6. Evaluation of DES Reusability

The recyclability of ChCl–U DES was evaluated under the optimized extraction conditions to explore the practical applicability of the UAE–DES process. After each extraction cycle, the DES fraction was recovered by rotary evaporation and reused directly without further purification, as described in Section 3.2.7.

**Figure 10:**
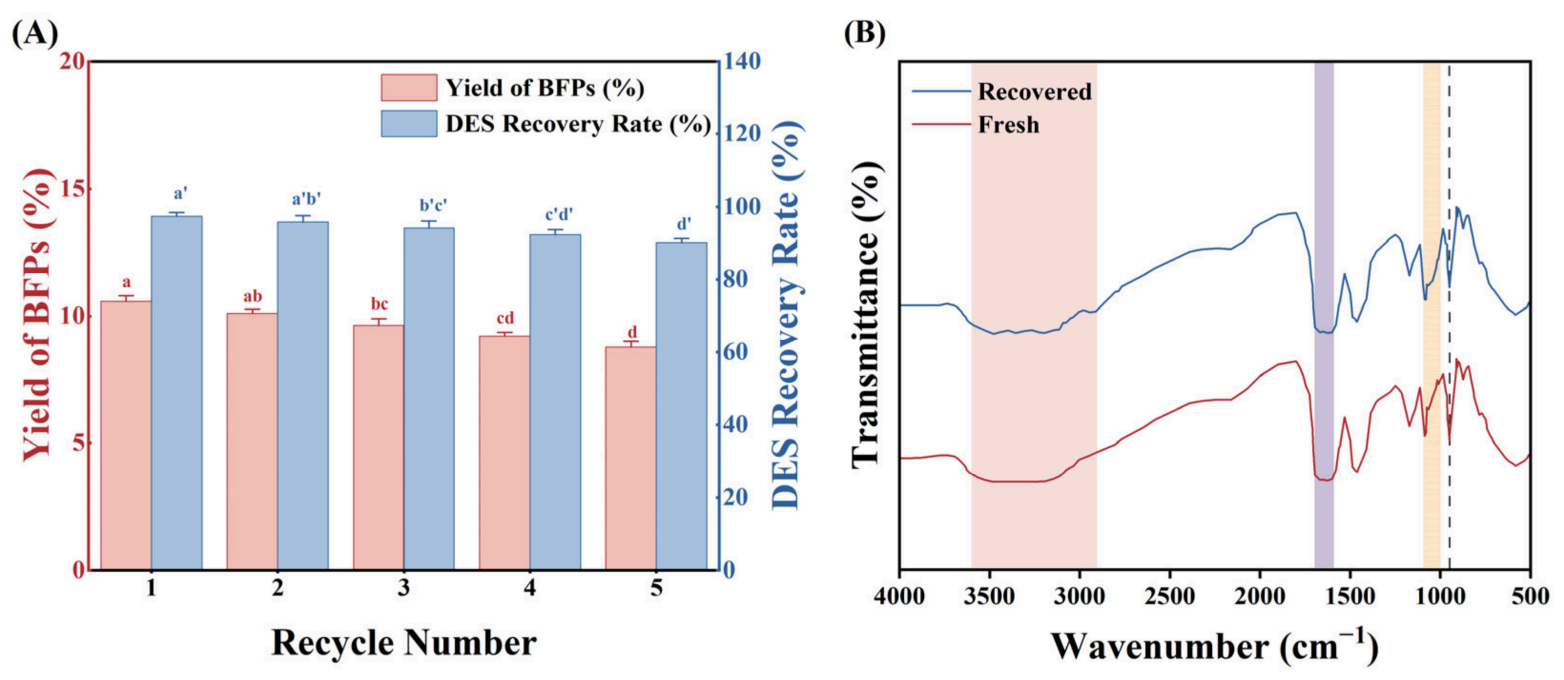
Recyclability of ChCl–U DES over five cycles. (**A**) Variation in BFP yield and DES recovery rate during five recycling cycles (n = 3). (**B**) FTIR spectra of fresh DES and recovered DES after five recycling cycles.

The results from five successive extraction recovery cycles revealed that the BFP yield decreased gradually from 10.58% to 8.78%, retaining 83.0% of the initial extraction capacity. Meanwhile, DES maintained a high recovery rate, declining from 97.30% to 90.20%, which indicates low solvent loss during the evaporation-based recovery procedure (Figure 10A). To further characterize the extraction medium and verify its stability during recycling, the physicochemical properties of the fresh and recovered DES were determined (Table 7). The freshly prepared ChCl–U DES exhibited a pH of 9.8 and a viscosity of 10.4 mPa·s at 25 °C. After five cycles, the viscosity of the recovered DES increased to 12.8 mPa·s, and the pH declined to 8.7. The water content of both DES samples was confirmed by Karl Fischer titration as 35% (*w*/*w*). Compared with the fresh DES, the FT-IR spectrum of the recovered DES, shown in Figure 10B, showed distinct changes in specific regions. These included a significantly enhanced absorption in the 1000–1100 cm−1 region, characteristic of the C–O–C and C–O stretching vibrations of the pyranose ring, indicating residual polysaccharide accumulation. Additionally, there was a broadened carbonyl absorption around 1600–1750 cm^−1^, which is attributable to the overlap of uronic acid carboxylate –COO^−^ with urea C=O stretching. We also observed an obvious enhancement in the C–H stretching band near 2900 cm^−1^ and a less-ordered O–H/N–H absorption in the 3000–3600 cm^−1^ region, reflecting a perturbation of the hydrogen-bond network. It is worth noting that the core DES skeleton vibration at 963 cm^−1^ remained highly stable. These observations provide direct evidence for the progressive accumulation of impurities within the DES medium during recycling. Accordingly, the gradual decline in extraction performance presumably arose from such impurity accumulation, which increased mass transfer resistance and partially disrupted the hydrogen-bond network. Even with these adverse effects, more than 80% of extraction efficiency was preserved after five cycles, demonstrating the favorable reusability of ChCl–U for BFP extraction.

### 2.7. Surface Morphology Analysis of Extraction Residues

**Figure 11:**
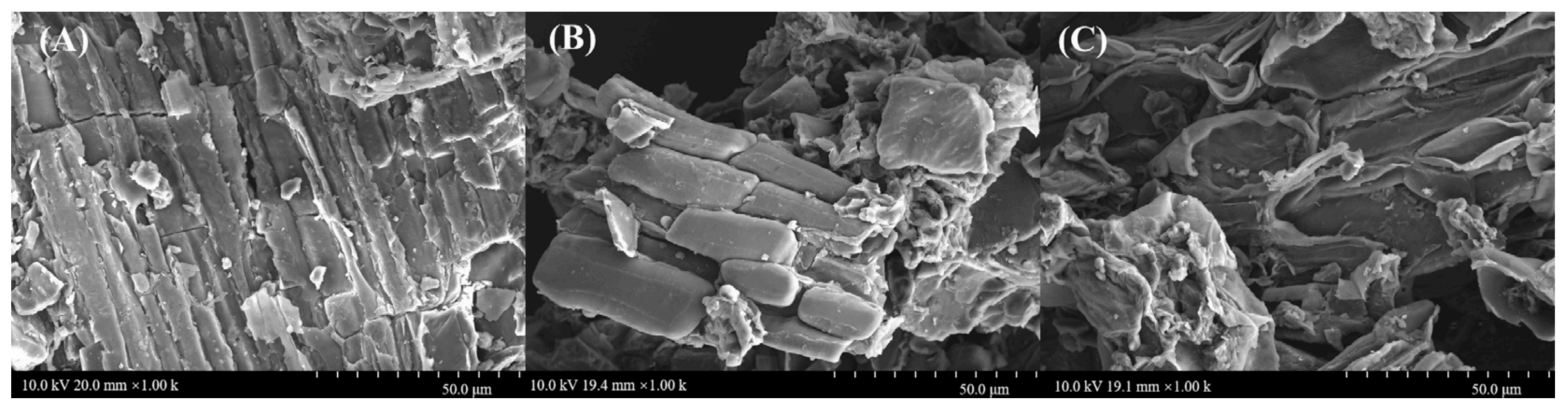
SEM micrographs of B. frutescens residues at 1000× magnification. (**A**) Untreated raw powder, (**B**) HWE residue, (**C**) UAE–DES residue.

Scanning electron microscopy (SEM) was employed to characterize the microstructural alterations in *B. frutescens* biomass induced by different extraction methods. Three groups of samples were prepared for comparative observation, including untreated raw powder as the blank control, residues from conventional hot water extraction (HWE), and solid residues obtained under optimized UAE–DES conditions. All micrographs were acquired at a magnification of ×1000. As shown in Figure 11A, the untreated raw material exhibited a smooth and compact surface with intact cellular frameworks. Only sporadic native micro-fissures were visible, with no artificial cracks or large cavities on the tissue surface. The dense and integrated lignocellulosic structure acted as a physical barrier that restricted solvent penetration and the outward diffusion of intracellular polysaccharides, thereby limiting polysaccharide release during conventional extraction. In comparison, HWE residues presented only minor structural erosion (Figure 11B). Thermal treatment induced localized surface cracking, slight peeling and partial tissue tearing, while most cellular morphologies remained intact. Simple thermal hydration merely loosened intercellular adhesion and failed to effectively disrupt the lignocellulosic network, which was consistent with the relatively low extraction efficiency of the HWE method described above. By contrast, UAE–DES-treated residues displayed severe structural destruction and disorganized microarchitecture (Figure 11C). Extensive tissue fragmentation, prominent cell-wall curling and lamellar peeling were observed, together with abundant cavities and fragmented cell debris. Combined ultrasonic cavitation and ChCl–U solvation disrupted the original ordered cellular structure of plant material. Specifically, ultrasonic shock waves and micro-jets generated powerful mechanical shear forces to rupture rigid cell-wall fibers, while ChCl–U DES disrupted intermolecular hydrogen bonding within lignocellulose matrices and further loosened plant tissues [28]. The resulting highly porous microstructure greatly increased the solid–liquid contact area and reduced internal mass transfer resistance, providing direct microscopic evidence for the superior BFP yield of the UAE–DES synergistic system. The varying degrees of cell-wall disintegration observed in the three groups were highly consistent with the extraction performance of different methods. These morphological differences visually illustrate the combined destructive effect of ultrasonic cavitation and ChCl–U solvation on plant tissue structures, which fundamentally accounts for the significantly improved release efficiency of polysaccharides.

### 2.8. Chemical Composition Analysis of BFPs

The protein and uronic acid contents of BFPs obtained via UAE–DES and conventional hot water extraction (HWE) were comparatively analyzed. As presented in Table 8, the total carbohydrate contents of UAE–DES–BFPs and HWE–BFPs were 78.26% and 80.32%, respectively. The protein content of UAE–DES–BFPs was 2.12%, which was considerably lower than the 2.58% determined for HWE–BFPs. Ultrasonic cavitation effectively breaks the cross-linking between polysaccharide chains and bound proteins, facilitating component dissociation and improving the removal efficiency of protein impurities during subsequent Sevag deproteinization [29]. Low residual protein content is favorable for maintaining the biological performance of polysaccharides, as excessive protein contaminants may interfere with bioactive evaluation and weaken the inherent bioactivity of polysaccharides [30]. By comparison, the uronic acid content of UAE–DES–BFPs reached 6.24%, which was significantly higher than that of HWE–BFPs (2.76%). Polysaccharides rich in uronic acid generally exhibit stronger antioxidant and immunomodulatory activities, owing to the negatively charged carboxyl groups that enable efficient interactions with free radicals and cell surface receptors [31]. Such compositional enrichment can be attributed to the mild extraction conditions and the selective solvation capability of DES for acidic polysaccharide fractions, which is consistent with previous plant polysaccharide studies [32]. Overall, the simultaneously reduced protein impurities and elevated uronic acid levels, alongside the comparable total carbohydrate contents, demonstrated that the optimized UAE–DES strategy not only achieves a higher extraction yield but also effectively improves the compositional quality of the obtained BFPs.

### 2.9. Structural Characterization of BFPs

#### 2.9.1. FT-IR Analysis

FT-IR spectra of HWE–BFPs and UAE–DES–BFPs are presented in Figure 1B. The broad absorption near 3439.2 cm^−1^ is associated with O–H stretching vibrations, whereas the low-intensity peak at 2928.5 cm^−1^ is characteristic of C–H stretching from alkyl substituents [33]. Bands at 1741.2 cm^−1^ and 1619.5 cm^−1^ originate from C=O stretching belonging to esterified carboxyl and deprotonated carboxylate groups of polysaccharide chains, which points to the occurrence of uronic acid moieties. The 1416.6 cm^−1^ band represents aliphatic C–H bending deformation; the weak feature at 1238.8 cm^−1^ also derives from C–H bending modes. The strong diagnostic band peaking at 1018.1 cm^−1^ originates from superimposed absorptions of C–O–C glycosidic bonds and C–O–H bending vibrations [34]. Moreover, peaks at 835.5 cm^−1^ and 916.7 cm^−1^ suggest that both *α*- and *β*-glycosidic configurations co-exist in BFPs.

No additional characteristic peaks assignable to DES components were observed in the spectrum of UAE–DES–BFPs. This observation suggests that DES functions merely as an extraction aid and does not introduce covalent modification or persistent solvent contamination onto polysaccharide backbones [35]. Despite highly similar peak positions for the two polysaccharide samples, obvious differences in band intensity, particularly for carboxyl-related signals, can be distinguished. Such spectral variation supports the higher uronic acid content of UAE–DES–BFPs determined in Section 2.8. These intensity differences reflect the intrinsic structural divergence between the two polysaccharide fractions. Such structural variations may alter their three-dimensional conformations, which could further influence interactions with biological receptors and thereby exert an impact on their biological properties.

#### 2.9.2. Molecular Weight Distribution

**Figure 12:**
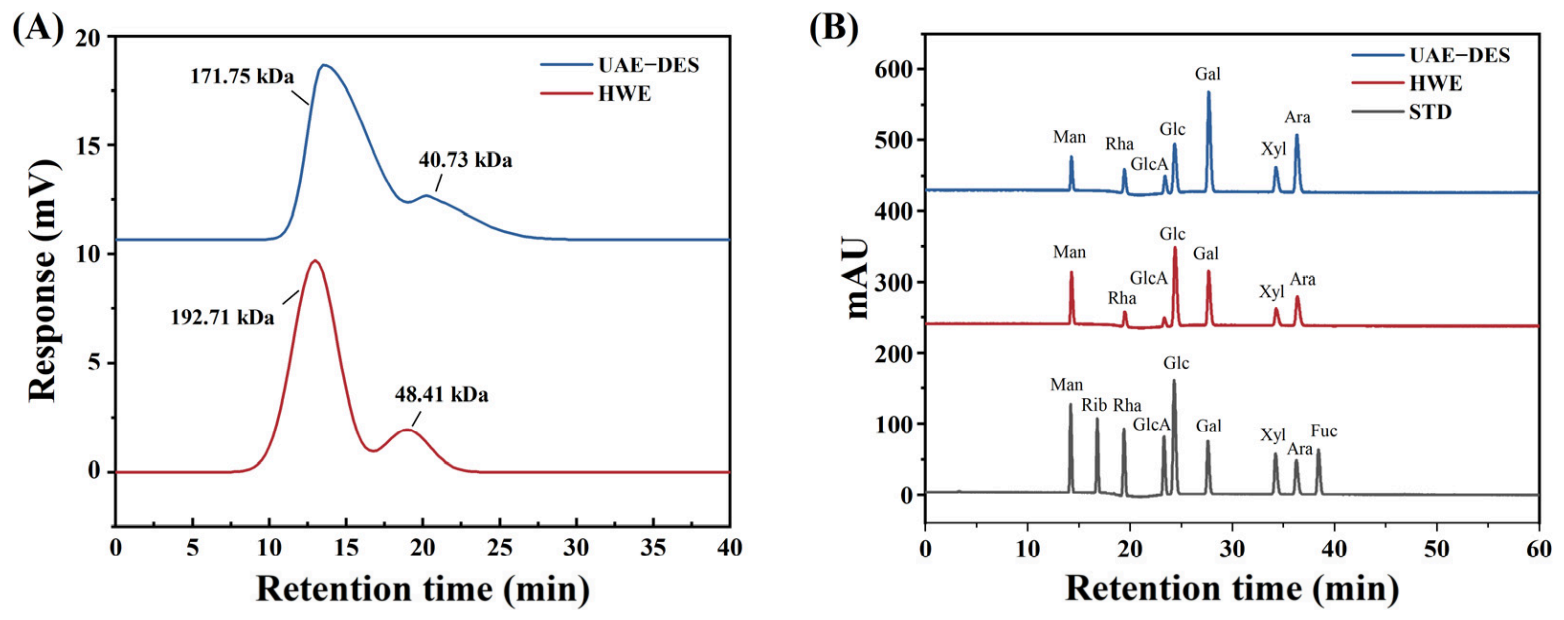
Spectral structural characterization of HWE–BFPs and UAE–DES–BFPs. (**A**) HPGPC molecular weight distribution curves, (**B**) HPLC profile of monosaccharide composition.

The molecular weight distributions of UAE–DES–BFPs and HWE–BFPs are displayed in Figure 12A. Both samples present two distinct elution peaks, demonstrating that BFPs consisted of two polysaccharide fractions with different molecular weights. The Mw values of Peak 1 and Peak 2 for HWE–BFPs were 192.71 kDa and 48.41 kDa, respectively, while those of UAE–DES–BFPs were 171.75 kDa and 40.73 kDa. Compared with HWE–BFPs, the molecular weights of both fractions in UAE–DES–BFPs were slightly lower, which was primarily caused by ultrasonic cavitation. The mechanical shear force generated during ultrasonic treatment could partially cleave polysaccharide glycosidic bonds, thereby inducing mild depolymerization of polysaccharide chains [36]. The proportion of the low-molecular-weight fraction increased from 21.2% for HWE-BFPs to 25.3% for UAE–DES–BFPs (Table 9), further verifying the occurrence of ultrasonic-induced polysaccharide depolymerization. The appropriately reduced molecular weight, together with well-preserved molecular homogeneity, could effectively improve the solubility and bioavailability of polysaccharides, endowing UAE–DES–BFPs with better application potential.

#### 2.9.3. Monosaccharide Composition

Monosaccharide profiles of UAE–DES–BFPs and HWE–BFPs were qualitatively and quantitatively determined by HPLC following PMP derivatization (Figure 12B). As summarized in Table 8, both samples contained the same seven monosaccharide constituents: mannose (Man), glucose (Glc), rhamnose (Rha), galacturonic acid (GalA), galactose (Gal), xylose (Xyl), and arabinose (Ara), while obvious discrepancies existed in their molar proportions. The shared monosaccharide types suggest that UAE–DES extraction does not change the fundamental monosaccharide constitution of BFPs [37]. Nevertheless, considerable differences in monosaccharide molar ratios were observed between the two extracts. Relative to HWE–BFPs, UAE–DES–BFPs showed elevated molar fractions of Gal and Ara, alongside decreased proportions of Glc and Man. Specifically, the molar percentage of Gal rose from 21.33% (HWE–BFPs) to 31.90% (UAE–DES–BFPs), and Ara increased from 14.01% to 22.23%. By contrast, the molar ratios of Glc and Man dropped from 34.58% to 17.35% and from 14.53% to 7.82%, respectively. These compositional differences indicate that UAE–DES enables selective enrichment of certain monosaccharide moieties, particularly Gal and Ara, which are typical building blocks of pectic-type polysaccharides [38]. Such selective accumulation of Gal and Ara in UAE–DES–BFPs may be attributed to the distinctive solvation behavior of DES. Strong hydrogen bond interactions between DES and plant polysaccharide networks can break weak glycosidic linkages within cell-wall matrices, favoring the preferential release of pectic fragments rich in Gal and Ara [39]. Furthermore, the hydrogen-bond-rich solvation microenvironment originating from DES constituents may induce the moderate, selective hydrolysis of cell-wall polysaccharides, which further contributes to the enrichment of pectin-derived monosaccharides in the final extracts [40].

In addition, UAE–DES–BFPs displayed higher uronic acid proportions than HWE–BFPs, in good agreement with the compositional data presented in Section 2.8. This cross-validation again confirms the capacity of UAE–DES to enrich acidic polysaccharide fractions. The existing literature reports that polysaccharides abundant in Gal, Ara and uronic acid frequently exhibit favorable antioxidant and immunomodulatory performance. Therefore, the altered monosaccharide profile of UAE–DES–BFPs may confer enhanced potential for biological activities [41]. Taken together, compared with conventional HWE, the UAE–DES approach not only enhances BFP extraction efficiency but also favorably modulates monosaccharide composition, offering a feasible strategy for obtaining polysaccharides with promising bioactive prospects.

#### 2.9.4. Thermogravimetric Analysis

**Figure 13:**
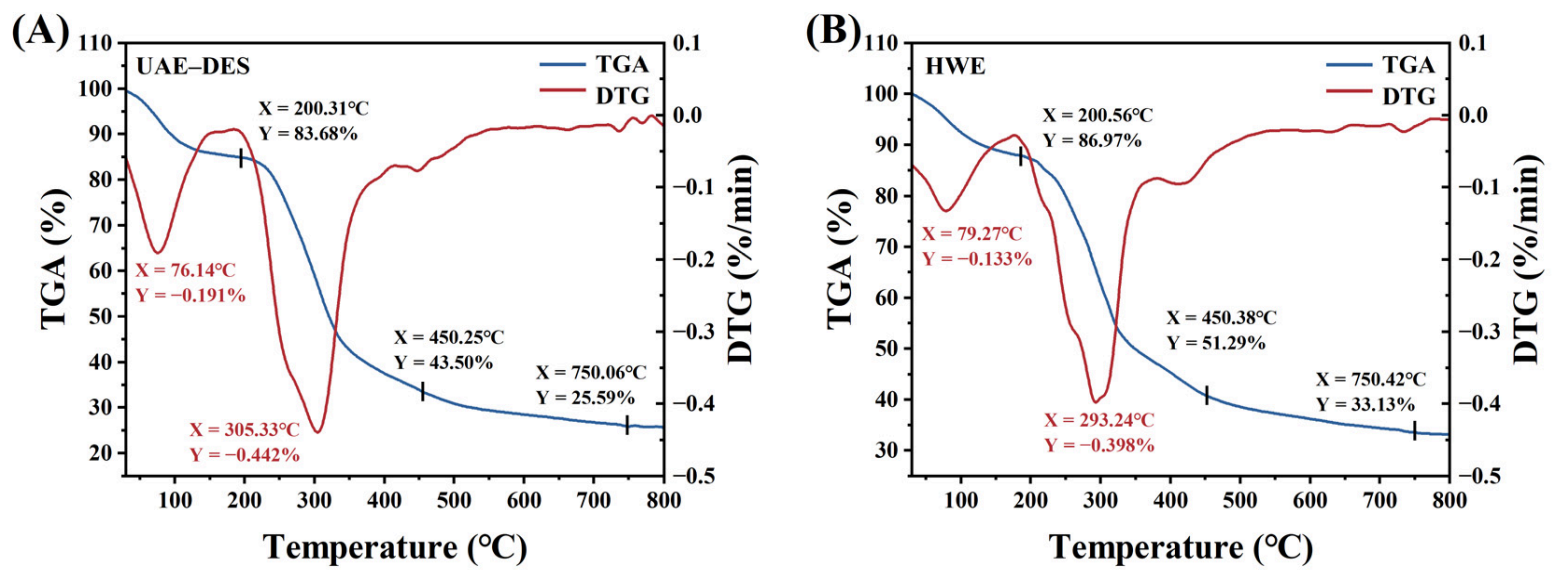
Thermal analysis of BFPs obtained from different extraction methods. (**A**) UAE–DES, (**B**) HWE.

Preserving the thermal stability of polysaccharides is essential for retaining their conformational integrity and bioactive characteristics during high-temperature processing and storage [42]. Thermogravimetric analysis was performed to monitor the physicochemical transitions of BFPs prepared by UAE–DES and HWE, including dehydration, backbone decomposition, and carbonization. As illustrated in Figure 13, both samples underwent three distinguishable mass-loss stages over the scanned range of 30–800 °C, with the corresponding derivative curves (DTG) shown alongside.

The initial weight-loss event, occurring from 30 to approximately 200 °C, was dominated by the removal of adsorbed and hydrogen-bonded water molecules [43]. UAE–DES–BFPs lost 16.32% of its mass in this interval, whereas HWE–BFPs exhibited a relatively lower loss of 13.03%. The DTG profiles located the maximum dehydration rates at 76.14 °C (UAE–DES–BFPs, −0.191%/min) and 79.27 °C (HWE–BFPs, −0.133%/min), respectively. The more pronounced water release from UAE–DES–BFPs may have arisen from the porous microstructure and enlarged surface area created by acoustic cavitation, which enhances moisture uptake and subsequent liberation upon heating.

The subsequent decomposition event (200–450 °C) represented the major thermal degradation phase, during which inter- and intra-chain hydrogen bonds were disrupted, glycosidic linkages were cleaved, and the polysaccharide backbone underwent depolymerization, accompanied by functional group transformation [44]. The mass reductions in this stage reached 40.18% for UAE–DES–BFPs and 35.68% for HWE–BFPs. The temperature at which the degradation rate attained its maximum (T_max_), as identified from the DTG minima, was 305.33 °C for UAE–DES–BFPs and 293.24 °C for HWE–BFPs, with peak decomposition rates of −0.442 and −0.398%/min, respectively. The 12 °C elevation in T_max_ observed for UAE–DES–BFPs signifies its greater resistance to thermal decomposition, since a higher temperature is required to trigger the most rapid chain scission. It is also worth noting that the DTG peak of HWE-BFPs was noticeably broader than that of UAE–DES–BFPs, consistent with its wider molecular weight distribution and higher protein content, which introduce a wider range of decomposition temperatures. In contrast, the sharper and more symmetric DTG peak of UAE–DES–BFPs reflects a more homogeneous population of polysaccharide chains with fewer impurities.

The final stage (450–800 °C) corresponded to the carbonization and oxidative consumption of the remaining organic skeleton. Mass losses of 17.91% and 18.16% were recorded for UAE–DES–BFPs and HWE–BFPs, respectively, in this interval. Beyond 600 °C, the mass decay tapered off and approached a plateau, leaving residual char yields of 25.59% and 33.13% at 750 °C. The higher char retention of HWE-BFPs is more likely associated with its elevated protein and inorganic impurity levels, which yield more carbonaceous and mineral residues upon pyrolysis, rather than indicating superior thermal resistance.

Collectively, the TGA–DTG results indicate that UAE–DES–BFPs possess enhanced thermal stability relative to HWE–BFPs, as demonstrated by its higher T_max_ and narrower decomposition peak. These discrepancies can be ascribed to the differences in molecular weight dispersity, protein content, and structural uniformity imparted by the two extraction approaches.

### 2.10. Anti-Complementary Activities of BFPs

**Figure 14:**
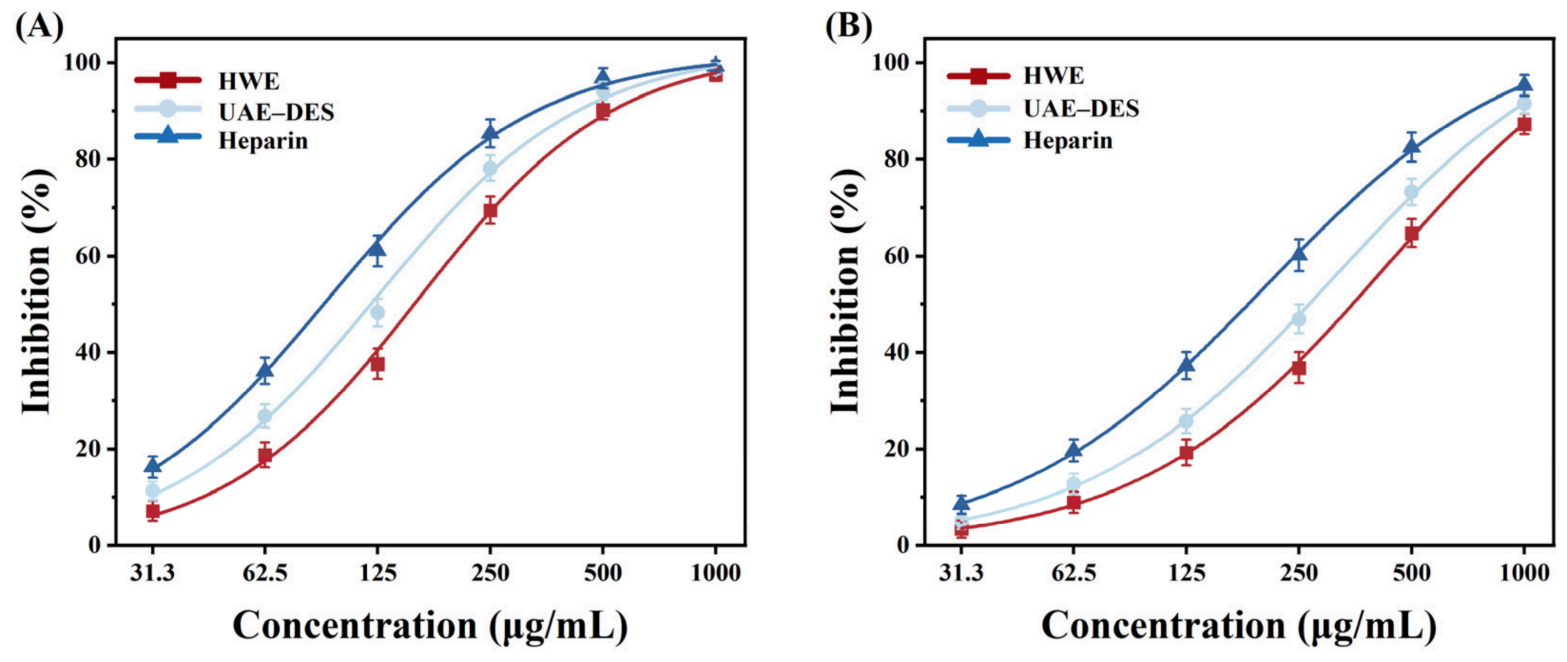
Dose–response curves for anti-complementary activities of BFPs. (**A**) Classical pathway, (**B**) alternative pathway. Heparin was used as positive control (n = 3).

The anti-complementary activities of BFPs prepared by UAE–DES and conventional HWE were evaluated via classical pathway (CH50) and alternative pathway (AP50) hemolytic assays, with heparin employed as positive control. As shown in Figure 14, both UAE–DES–BFPs and HWE–BFPs exerted concentration-dependent inhibitory effects on the classical and alternative complement pathways. Within the tested concentration range (31.3–1000 μg/mL), the hemolytic inhibition rate increased gradually with elevated polysaccharide dosage. Notably, UAE–DES–BFPs exhibited stronger anti-complementary potency than HWE–BFPs, as evidenced by lower CH_50_ and AP_50_ values. Specifically, the CH_50_ values of UAE–DES–BFPs and HWE–BFPs were 120.13 ± 2.26 μg/mL and 156.70 ± 3.75 μg/mL, while their corresponding AP_50_ values were 255.54 ± 3.12 μg/mL and 332.46 ± 2.38 μg/mL, respectively. As the positive control, heparin showed superior inhibitory activity, with CH_50_ and AP_50_ values of 89.59 ± 1.84 μg/mL and 177.15 ± 2.43 μg/mL. Given the comparable total carbohydrate contents of the two extracts (a difference of <3%), the mass-based concentrations provided a reliable basis for the relative comparison of their anti-complementary activities.

The improved anti-complementary performance of UAE–DES–BFPs can be reasonably interpreted by their compositional characteristics verified in previous analyses. The higher uronic acid content provides abundant negatively charged carboxyl groups, which can interact with positively charged complement components and block the overactivation of the complement cascade [45]. Furthermore, the elevated proportions of galactose and arabinose in UAE–DES–BFPs also contribute to enhanced bioactivity, consistent with previous findings that these monosaccharide moieties are closely associated with favorable anti-complementary capacity [46]. Such activity differences demonstrated that the UAE–DES extraction strategy not only improves polysaccharide extraction efficiency but also effectively optimizes the bioactivity of BFPs. Unlike conventional hot water extraction, which tends to cause non-selective component dissolution, the UAE–DES system achieves selective enrichment in bioactive polysaccharide fractions. Accordingly, the obtained UAE–DES–BFPs possess prominent anti-complementary potential, which makes them promising natural inhibitors for relieving complement-mediated inflammatory disorders [47].

### 2.11. Identification of Complement Components Involved in Inhibitory Effects

To further uncover the molecular mechanism responsible for the anti-complementary potency of BFPs, complement-depleted human sera were utilized to screen the complement components that participate in the inhibitory effect of both UAE–DES–BFPs and HWE–BFPs. Classical pathway hemolytic assays were conducted using sera individually deficient in C1q, C2, C3, C4, C5, and C9. In this assay system, sustained hemolysis inhibition means that this complement component participates in the inhibitory action of BFPs, while obvious hemolysis recovery implies that the component is not involved in the polysaccharide-induced inhibitory process.

**Figure 15:**
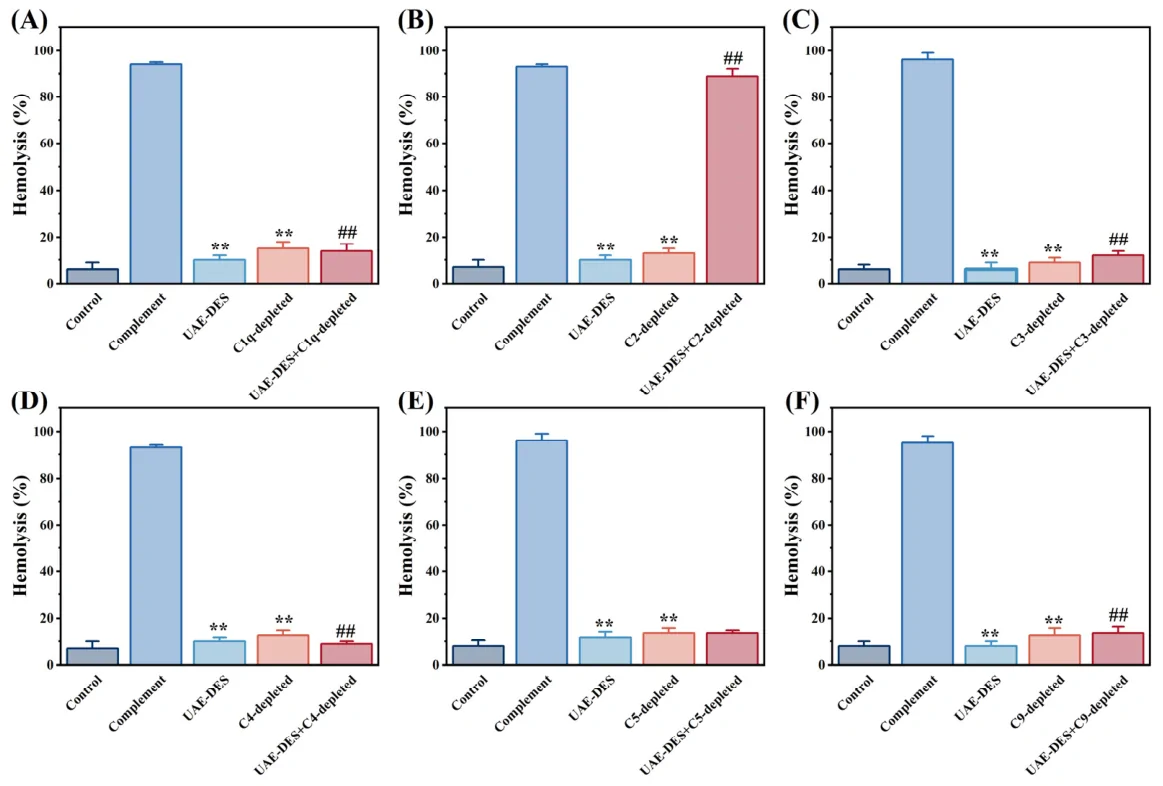
Complement components involved in anti-complement effect of UAE–DES–BFPs (400 μg/mL). (**A**–**F**) C1q, C2, C3, C4, C5 and C9, respectively (n = 3). ** *p* < 0.01, compared with complement group; ## *p* < 0.01, compared with corresponding complement-depleted group.

**Figure 16:**
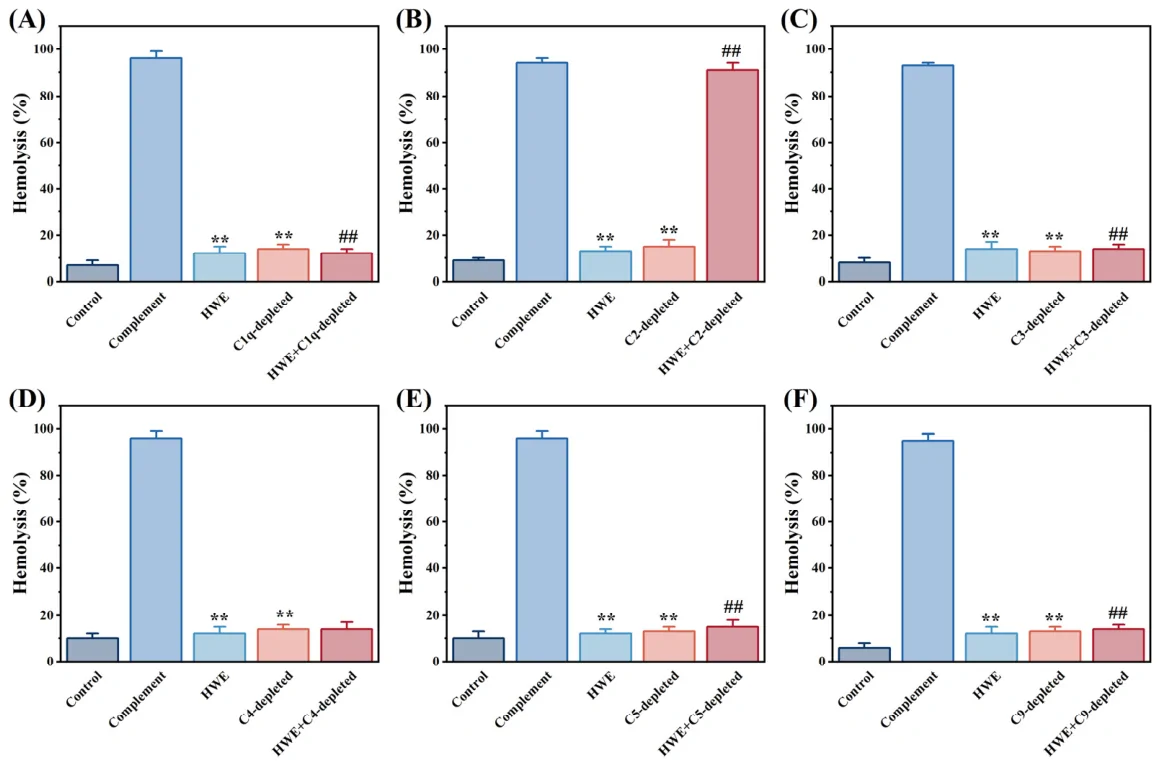
Complement components involved in anti-complement effect of HWE–BFPs (400 μg/mL). (**A**–**F**) C1q, C2, C3, C4, C5 and C9, respectively (n = 3). ** *p* < 0.01, compared with complement group; ## *p* < 0.01, compared with corresponding complement-depleted group.

As presented in Figure 15 and Figure 16, the inhibitory-relevant complement components of the two BFPs were screened using complement-depleted sera. None of the complement-depleted sera independently lysed erythrocytes, with hemolytic percentages below 15%. After treatment with the BFPs, hemolysis was not restored in the C1q-, C3-, C4-, C5-, and C9-depleted sera, whereas obvious hemolysis recovery was observed in the C2-depleted serum, with recovery rates of 89.3 ± 3.1% and 91.0 ± 2.8% for UAE–DES–BFPs and HWE–BFPs, respectively. These results indicated that both BFPs block the activation cascade of the complement system, acting through C1q, C3, C4, C5, and C9 but not C2.

Notably, although both BFPs rely on the same set of complement components to exert their inhibitory effects, UAE–DES–BFPs possessed significantly stronger anti-complementary activity. This disparity indicated that the extraction method influences the inhibitory potency rather than which complement components are engaged, and the enhanced activity of UAE–DES–BFPs was consistent with their favorable compositional characteristics discussed in the preceding section. However, the precise structure–activity relationship and the molecular mechanism of BFPs require further investigation.

### 2.12. Inhibition of Complement C3 and C4 Deposition

**Figure 17:**
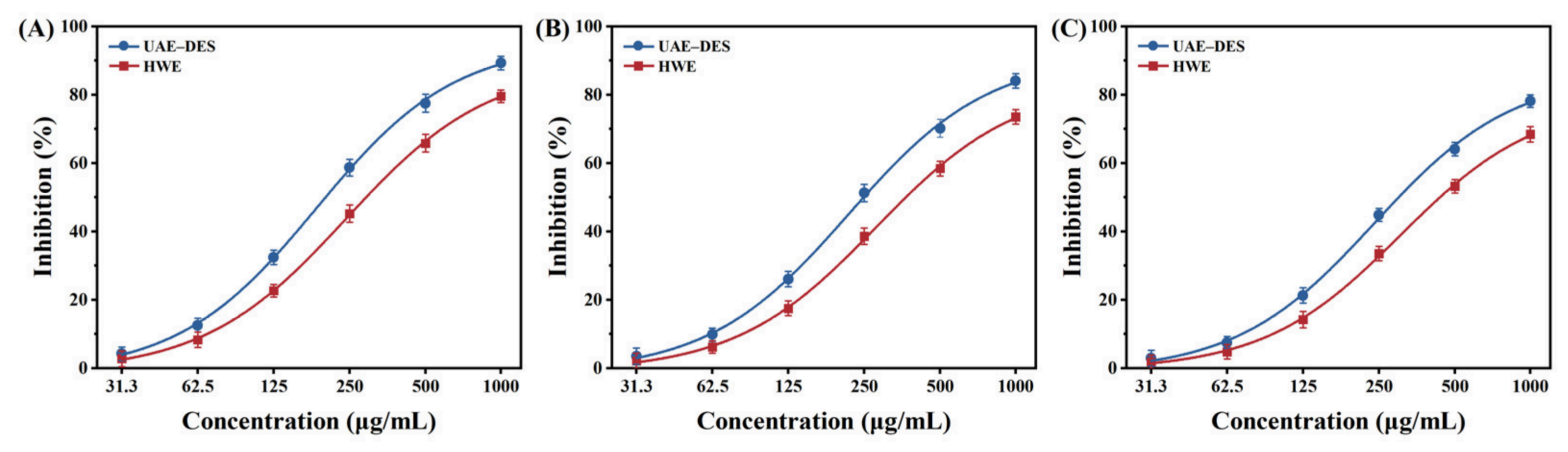
Inhibitory effects of BFPs on complement C3 and C4 deposition. (**A**) C3 deposition in the classical pathway, (**B**) C4 deposition in the classical pathway, and (**C**) C3 deposition in the alternative pathway (n = 3).

To further delineate the mechanism underlying the anti-complementary activity of BFPs, the effects of UAE–DES–BFPs and HWE–BFPs on complement C3 and C4 deposition were quantified by an enzyme-linked immunosorbent assay. Both samples suppressed the deposition of C3 and C4 on the activated microtiter well surface in a concentration-dependent manner, with UAE–DES–BFPs consistently exhibiting greater inhibitory potency than HWE–BFPs across all tested conditions (Figure 17). In the classical pathway, UAE–DES–BFPs at 250 μg/mL reduced C3 deposition by 58.72 ± 2.13% and C4 deposition by 51.36 ± 1.95%, whereas HWE-BFPs at the same concentration achieved 45.25 ± 1.87% and 38.64 ± 1.62% inhibition, respectively. The IC_50_ for C3 deposition were 184.37 ± 6.82 μg/mL for UAE–DES–BFPs and 241.93 ± 8.15 μg/mL for HWE–BFPs, while the corresponding values for C4 deposition were 216.52 ± 7.46 and 277.69 ± 9.28 μg/mL. In the alternative pathway, UAE–DES–BFPs also displayed stronger suppression of C3 deposition than HWE–BFPs, with inhibition rates of 44.87 ± 1.76% versus 33.51 ± 1.43% at 250 μg/mL and IC_50_ values of 240.94 ± 8.73 and 303.51 ± 10.62 μg/mL, respectively. These results are in good agreement with the CH_50_ and AP_50_ data and confirm that BFPs interfere with the activation and covalent deposition of C3 and C4, which represent the central convergence points of both complement pathways. The observation that C3 deposition was more strongly inhibited than C4 deposition is consistent with the enzymatic amplification role of C3 convertase, as reduced C4 cleavage directly limits the formation of classical pathway C3 convertase and thereby attenuates downstream C3 activation [48]. Combined with the screening results showing C1q, C3, C4, C5, and C9 as components participating in the inhibitory response, these findings suggest that UAE–DES–BFPs attenuate complement activation at multiple stages, with a particularly pronounced impact on the C3 and C4 convertase reactions.

## 3. Materials and Methods

### 3.1. Materials and Reagents

Aerial portions of *Baeckea frutescens* L. were purchased at a medicinal raw material market in Maoming, Guangdong Province, China. Species identification was completed by Dr. Jiaming Wang (College of Pharmacy, Qiqihar Medical University). A voucher specimen (No. QY-BF-2211) is preserved in the specimen repository. Detailed information regarding chemicals, sera, antibodies and assay reagents is provided in the Supplementary Materials.

### 3.2. Extraction and Optimization of BFPs

#### 3.2.1. Preparation of DESs

Eleven DES systems were prepared following a previously reported method [49]. Choline chloride as the hydrogen bond acceptor with various hydrogen bond donors at selected molar ratios (Table S1) [50,51,52]. Each mixture was heated at 80 °C with stirring until a clear homogeneous liquid was obtained. All DESs were stored in sealed vials at room temperature. The pH and viscosity of the optimized DES were measured using a pH meter (Accumet XL 20, Fisher Scientific, Waltham, MA, USA) and a rotational viscometer (Discovery HR-1 hybrid rheometer, TA Instruments, New Castle, DE, USA) at 25 °C. The water contents of the freshly prepared and the recovered DES were determined by Karl Fischer titration, using an 831 KF Coulometer with a generator electrode without a diaphragm (Metrohm, Herisau, Switzerland). HYDRANAL-Coulomat AG (Honeywell, Charlotte, NC, USA) was used as an anolyte solution. Results are expressed as weight percentages (*w*/*w*). The FT-IR spectrum of the DES was recorded in ATR mode over 4000–400 cm^−1^ using an FT-IR spectrometer (Thermo Scientific Nicolet iS50, Thermo Scientific, Madison, WI, USA).

#### 3.2.2. DES Screening and Comparison with Conventional Water Extraction

Dried powder of *B. frutescens* was ground and passed through a 60-mesh sieve. The obtained powder underwent two rounds of defatting treatment using petroleum ether at a solid-to-solvent ratio of 1:10 (*w*/*v*), followed by overnight air drying. For UAE–DES extraction, 1 g of pre-defatted powder was dispersed in 20 mL of DES within a stoppered Erlenmeyer flask. A Scientz-1200E ultrasonic processor (Ningbo Scientz Biotechnology Co., Ltd., Ningbo, China) fitted with a 20 mm titanium probe was operated at a working frequency of 20 kHz. The ultrasound was applied intermittently (9 s on, 1 s off), and a circulating water bath was connected to maintain the target extraction temperature. The probe tip was submerged approximately 3–4 cm below the liquid surface and offset from the flask wall to promote uniform cavitation and heat dissipation. The extraction was carried out at 30% (*w*/*w*) water content, 60 °C, 150 W power output, and 60 min contact time. A parallel ultrasonic-assisted water extraction (UAE–W) was performed under identical conditions except that distilled water replaced DES. Hot water extraction, serving as the conventional control, was implemented at 100 °C over a 2 h period in the absence of sonication under identical solid-to-solvent proportions. After extraction, centrifugation of the mixture at 4000 rpm for 15 min yielded the supernatant fraction. Four volumes of anhydrous ethanol were introduced to precipitate crude polysaccharides, and the mixture was kept at 4 °C for 12 h. The precipitated fraction obtained by centrifugation was redissolved in deionized water and subjected to five Sevag deproteinization cycles (chloroform/*n*-butanol, 4:1, *v*/*v*) [53]. Using a 3500 Da cutoff membrane, the aqueous layer was dialyzed in distilled water for 48 h, and purified BFPs were obtained after concentration and lyophilization. Each experiment was completed in triplicate.

Total sugar content was determined via the phenol-sulfuric acid assay, with glucose selected as the reference standard, and the yield is expressed as glucose equivalents [54]. The resulting calibration function is expressed as Y = 5.1268X + 0.1437 (R^2^ = 0.9991), where Y corresponds to absorbance recorded at 490 nm, and X denotes glucose concentration. The polysaccharide extraction yield was computed using:

Y = (C × V × X)/M × 100% (1)

where Y denotes the polysaccharide extraction yield (%); C represents the glucose concentration (mg/mL) derived from the absorbance reading of the standard curve; V is the volume of polysaccharide aqueous solution (mL); X refers to the dilution factor; M stands for the mass of the *B. frutescens* starting material (mg).

#### 3.2.3. Single-Factor Experimental Design

Single-factor experiments were first conducted to delineate the favorable operating ranges of the principal variables prior to RSM optimization. The influence of five independent parameters on BFP yield was evaluated, including DES water content (10–50%, *w*/*w*), liquid–solid ratio (5–40 mL/g), ultrasonic temperature (30–70 °C), ultrasonic power (50–250 W), and extraction time (20–60 min). Each parameter was tested stepwise across its designated levels while the remaining four were held at fixed baseline conditions. The baseline was set as 30% water content, 20 mL/g liquid–solid ratio, 60 °C, 150 W, and 60 min extraction time. Once the optimal level of one variable was identified, it was adopted as the fixed baseline for the subsequent parameter. All experiments were performed in triplicate, and the resulting data were used to establish the basis for the following BBD.

#### 3.2.4. RSM Optimization

Based on the single-factor results, a BBD with four independent variables (DES water content, ultrasonic temperature, ultrasonic time, and liquid–solid ratio) at three levels was generated by Design-Expert 13 software. The factor levels are listed in Table S2. The model significance was evaluated by ANOVA, and the goodness of fit was assessed by R^2^ and adjusted R^2^. Three-dimensional response surface plots were constructed to visualize factor interactions.

The experimental data were fitted to a second-order polynomial model to express the response (extraction yield of BFPs) as a function of the independent variables. The generalized model is represented by the following Equation (2):

(2) $$Y\;=\beta_{0}\;+\;\sum_{i=1}^{4}\beta_{i}X_{i}\;+\;\sum_{i=1}^{4}\beta_{\mathrm{ii}}{X_{i}}^{2}+\;\sum_{i=1}^{4}\sum_{j<i}^{4}\beta_{\mathrm{ij}}X_{i}X_{j}$$

where Y represents the predicted response value; *β*_0_ denotes the constant term; *β*_i_, *β*_ii_, and *β*_ij_ correspond to the coefficients of linear, quadratic and interaction terms, respectively; while X_i_ and X_j_ are coded independent variables.

#### 3.2.5. GA–BP Neural Network Modeling and Comparison with RSM

A three-layer back-propagation (BP) neural network was built in MATLAB R2024b using the 29 RSM datasets. The network consisted of four input neurons (the four process variables), ten hidden neurons (selected based on preliminary trials on the training set), and one output neuron (BFP yield). The input variables were normalized to the range of −1 to 1 using the mapminmax function before training. The datasets were randomly divided into training (70%), validation (15%), and testing (15%) subsets. The random seed was fixed for reproducibility. The Levenberg–Marquardt algorithm (trainlm) was adopted to train the neural network. The hidden layer employed the tan-sigmoid transfer function, whereas the output layer utilized the pure-linear transfer function [55]. Training was configured with a maximum of 1000 epochs and a performance goal of 1 × 10^−5^, and terminated early when the validation error failed to decrease for six consecutive epochs. For the hybrid GA–BP model, the genetic algorithm was implemented to optimize the initial weights and thresholds. The population size was set as 50, with a crossover probability of 0.8 and a mutation probability of 0.05. Model performance was evaluated on the training, validation, and test subsets using the R^2^, RMSE, and MAE. Leave-one-out cross-validation was additionally performed to obtain a more robust out-of-sample performance estimate, and the results were compared with those obtained from RSM.

(3) $$R^{2}=1-\sum_{i=1}^{n}\frac{{\left(y_{i}-y_{x}\right)}^{2}}{{\left(y_{i}-y_{z}\right)}^{2}}$$

(4) $$\mathrm{RMSE}=\sqrt{\frac{1}{n}\sum_{i=1}^{n}{\left(y_{i}-y_{x}\right)}^{2}}$$

(5) $$\mathrm{MAE}=\frac{1}{n}\sum_{i=1}^{n}\left|y_{i}-y_{x}\right|$$

#### 3.2.6. Extraction Kinetics Analysis

Kinetic extraction tests were performed under ultrasonic power of 200 W, covering three temperature levels (303, 313, and 323 K) and three liquid–solid ratios (10:1, 20:1, and 30:1 mL/g). Small sample aliquots were collected in 5 min time intervals throughout the 60 min extraction run for quantification of polysaccharide concentration. The acquired experimental datasets were fitted to a kinetic model deduced from Fick’s second law for spherical particles [56]. Under the assumptions of uniform internal distribution and negligible external mass transfer resistance, the diffusion equation was solved by the Fourier series method, and the first term (n = 1) was retained to give Equation (6):

(6) $$\ln\left[\frac{C_{\infty }}{C_{\infty }-C_{t}}\right]=kt+\ln\frac{\pi^{2}}{6}$$

where C_t_ refers to the polysaccharide concentration measured at time t (mg/mL), C_∞_ represents the equilibrium concentration (mg/mL), and *k* denotes the extraction rate constant (min^−1^).

The relative residual rate (Y) was defined by Equation (7) and fitted to the exponential decay model to further validate the kinetic behavior:

(7) $$Y=\left(C_{\infty }-C_{t}\right)/C_{\infty }=A\cdot \exp^{\left(-kt\right)}$$

The apparent activation energy (E_a_) was determined from the Arrhenius equation:

*k* = A⋅exp(−E_a_/RT) (8)

where A denotes the pre-exponential factor, R represents the universal gas constant (8.314 J·mol^−1^·K^−1^), and T corresponds to absolute temperature in Kelvin (K). A linear plot of ln *k* versus 1/T was constructed, and the activation energy E_a_ was derived from the slope obtained via linear regression analysis of this dataset.

#### 3.2.7. DES Recyclability Assessment

The reusability of the optimized DES was evaluated over five consecutive extraction cycles [57]. After each cycle, the DES was recovered from the supernatant by rotary evaporation at 65 °C to remove ethanol and residual water, then reconstituted to the original water content (35%) and reused directly. The DES recovery rate and BFP yield were determined for each cycle to assess the retention of extraction performance. The DES recovery rate was determined using Equation (9).

(9) $$\mathrm{Recovery rate}=\frac{v_{1}}{v_{0}}\times 100\%$$

where V_1_ is the volume of the recovered DES after rotary evaporation and reconstitution to the original water content (35%, *w*/*w*) (mL), and V_0_ is the initial DES volume (mL) used for extraction.

In addition, to evaluate the stability of the DES during recycling, the pH, viscosity, and FT-IR spectrum of the fresh and recovered DES were measured as described in Section 3.2.1.

### 3.3. Surface Morphology Analysis of Extraction Residues by SEM

Morphological alterations of *B. frutescens* extraction residues were characterized via scanning electron microscopy (Hitachi SU8010, Tokyo, Japan). Raw untreated powder, HWE residue, and UAE–DES-treated residue were affixed onto aluminum stubs, gold-sputter-coated, and imaged under an accelerating voltage of 10 kV at 1000× magnification.

### 3.4. Chemical Composition Analysis

The total carbohydrate content of the polysaccharide samples was determined by the phenol-sulfuric acid method using glucose as the calibration standard, following the procedure described in Section 3.2.2. Protein content was quantified via the Bradford assay, with bovine serum albumin serving as the calibration standard [58]. The corresponding calibration curve was defined as Y = 0.4152X + 0.4683 (R^2^ = 0.9968), where Y represents absorbance recorded at 595 nm, and X denotes BSA concentration. Uronic acid content was assayed using the carbazole-sulfuric acid procedure, and galacturonic acid was adopted as the reference standard [59]; its calibration curve was Y = 2.0573X + 0.0794 (R^2^ = 0.9958) measured at 530 nm. Every measurement was carried out in biological triplicates.

### 3.5. Structural Characterization of BFPs

#### 3.5.1. FT-IR Analysis

FT-IR characterization was performed on a Nicolet iS50 spectrometer (Thermo Fisher Scientific, Madison, WI, USA). Solid pellets were prepared by homogenizing sample powder together with KBr matrix under compression. Spectral acquisition covered the wavenumber interval ranging from 4000 to 400 cm^−1^, with spectra collected at 4 cm^−1^ resolution over 32 repeated scans for signal averaging.

#### 3.5.2. Molecular Weight Distribution Determination

The molecular weight profiles of BFPs were characterized via HPGPC on an Agilent 1260 platform equipped with a refractive index detector and a TSKgel GMPwxl column (300 × 7.8 mm). Isocratic elution was performed using 0.2 M NaCl aqueous solution. The column compartment was maintained at 40 °C while the eluent was delivered at 0.7 mL/min. Molecular weight data were calculated relying on a calibration curve constructed from multiple dextran reference materials.

#### 3.5.3. Monosaccharide Composition Analysis

Monosaccharide composition was characterized by HPLC coupled with PMP pre-column derivatization [60]. Lyophilized BFP sample (5 mg) was hydrolyzed with 2 M trifluoroacetic acid at 110 °C for 6 h. The hydrolysate was dried under a nitrogen stream, reconstituted in aqueous solution and derivatized with PMP. After derivatization, the sample was centrifuged and filtered through a 0.22 μm membrane before injection. Chromatographic separation was performed on a Waters C18 column (250 × 4.6 mm, 5 μm) maintained at 35 °C. The mobile phase was phosphate buffer (pH 7.0)/acetonitrile (83:17, *v*/*v*) delivered at a flow rate of 1.0 mL/min, and the detection wavelength was set at 245 nm. Monosaccharides were identified by comparing retention times with reference standards and quantified by the external standard method using peak areas. The measured concentrations were converted to molar quantities to calculate the molar ratios of monosaccharide components.

#### 3.5.4. Thermogravimetric Analysis

Thermal degradation was analyzed on a Pyris Diamond TGA (PerkinElmer, Shelton, CT, USA). Around 5 mg of lyophilized sample was subjected to thermal analysis, where the temperature rose from 30 °C to 800 °C at a heating rate of 10 °C/min under continuous nitrogen purge at 50 mL/min. The derivative thermogravimetric (DTG) curve was computed to identify the temperature of maximum decomposition rate (T_max_), and the residual mass at each stage was quantified.

### 3.6. Complement Assay

#### 3.6.1. Complement Assay Through the Classical Pathway

The classical pathway activity was evaluated using sensitized sheep erythrocytes, as described previously [61]. Briefly, NHSP was diluted at a ratio of 1:80 using GVB^++^ buffer. The diluted serum solution was pre-incubated with serially gradient-diluted BFP samples at 37 °C for 15 min under gentle shaking. Afterwards, sensitized sheep erythrocyte suspensions were introduced to each reaction system, and incubation proceeded for another 30 min at the identical temperature. Cold physiological saline was added to quench the complement-mediated hemolysis reaction. Samples were then centrifuged to pellet cell debris, and the absorbance of collected supernatants was spectrophotometrically recorded at 540 nm. Heparin was included in parallel experimental wells as the positive inhibitory control. The CH_50_ value, representing the sample concentration producing 50% inhibition of total complement hemolytic activity, was calculated via nonlinear regression fitting.

#### 3.6.2. Complement Assay Through Alternative Pathway

For assessment of alternative complement pathway activity, NHSP was diluted 1:7 with GVB-Mg^2+^/EGTA buffer to suppress activation of the classical complement pathway. Gradient serially diluted BFP samples were pre-incubated with the diluted serum at 37 °C for 15 min under gentle shaking. Subsequently, rabbit erythrocyte suspensions were dispensed into each reaction mixture, followed by a further 30 min incubation at the same temperature. The hemolytic reaction was terminated by transferring samples onto an ice-bath. After centrifugation to pellet cell debris, the absorbance of the resulting supernatants was determined spectrophotometrically at 412 nm. The AP_50_ value was calculated accordingly.

#### 3.6.3. Complement Depletion Assay

Complement components participating in BFPs-mediated inhibition were determined using human complement-depleted sera according to a method described previously [61]. Briefly, BFPs were pre-incubated with NHSP at 37 °C for 10 min, then mixed with the indicated depleted serum and sensitized sheep erythrocytes. Five groups were set for each depleted serum: control, complement, BFPs, depleted serum group, and BFPs + depleted serum group. Hemolysis recovery in the BFPs + depleted serum group relative to the depleted serum group indicated that the corresponding component is not required for BFPs-mediated inhibition, whereas sustained hemolysis inhibition indicated that this component participates in the inhibitory process.

#### 3.6.4. Complement C3 and C4 Deposition Assay

The effects of BFPs on complement C3 and C4 deposition were evaluated by an enzyme-linked immunosorbent assay [62]. For the classical pathway assay, microtiter plates were coated with 10 µg/mL human IgM diluted in carbonate buffer (pH 9.6) and incubated overnight at 4 °C. After blocking with 1% BSA in PBS, diluted normal human serum (1:100 in GVB^++^ buffer) was added to each well in the presence or absence of serially diluted BFPs, followed by incubation at 37 °C for 30 min. For the alternative pathway, plates were coated with 50 µg/mL zymosan in PBS, and serum diluted 1:20 in GVB-Mg^2+^/EGTA buffer was incubated with BFPs under the same conditions. After washing with PBS containing 0.05% Tween-20, deposited C3 and C4 fragments were detected with horseradish-peroxidase-conjugated goat anti-human C3c and C4c antibodies, respectively. Color development was performed using TMB substrate, and the reaction was quenched with 2 M H_2_SO_4_. The absorbance was measured at 450 nm. The percentage inhibition of C3 or C4 deposition was calculated relative to the untreated control, and IC_50_ values were derived from dose–response curves.

### 3.7. Statistical Analysis

ANOVA was carried out with GraphPad Prism 8.0. Design-Expert 13 was utilized for the execution of the Box–Behnken design, while the GA–BP neural network was built within MATLAB R2024b. Origin 2021 was employed for graph plotting. Differences yielding *p* < 0.05 were regarded as statistically significant.

## 4. Conclusions

In this study, an ultrasound-assisted deep eutectic solvent extraction method was developed and optimized for the extraction of polysaccharides from *B. frutescens* using an integrated RSM and GA–BP framework. Under the optimized conditions, the BFP yield reached 10.58 ± 0.21%, representing a 4.4-fold increase compared with that achieved with conventional hot water extraction. The UAE–DES extraction yielded polysaccharide fractions with distinct monosaccharide compositions and slightly lower molecular weights compared with HWE–BFPs. Kinetic analysis based on Fick’s second law established a diffusion-controlled extraction mechanism with a low apparent activation energy, providing a theoretical basis for process scale-up, and the DES solvent retained over 83% of its extraction capacity after five recycling cycles, supporting the reusability of the extraction solvent.

UAE–DES–BFPs exhibited stronger anti-complementary activity than HWE–BFPs against both the classical and alternative complement pathways. Complement depletion assays revealed that both fractions blocked the complement activation cascade, acting through C1q, C3, C4, C5, and C9, and that the enhanced activity of UAE–DES–BFPs was traced to more potent inhibition of C3 and C4 deposition rather than a shift in the engaged complement component set. These findings demonstrate that extraction conditions influence not only the polysaccharide yield but also the structural composition and biological performance of the polysaccharides. Nevertheless, the bioactivity assessments were conducted exclusively through in vitro assays, and in vivo validation is required to confirm the therapeutic potential and safety of BFPs. Future studies should focus on in vivo efficacy and safety evaluations, process scalability, and detailed structural characterization of purified fractions to fully understand the structure–activity relationships. Taken together, the present results suggest that UAE–DES is an efficient approach for the extraction of polysaccharides from *B. frutescens*, and the obtained BFPs could be developed as potential natural complement inhibitors.

## Figures and Tables

**Table 1:** The actual and predicted data from the BBD and the predicted data from GA–BP.

Runs	A	B	C	D	Yield of BFPs (%)	BBD Predicted Value	GA–BP Predicted Value
1	40	40	30	30:1	8.03	8.01	8.03
2	20	40	30	30:1	7.74	7.79	7.78
3	20	50	30	40:1	8.26	8.31	8.27
4	20	60	30	30:1	8.07	8.12	8.07
5	30	60	30	20:1	9.43	9.38	9.45
6	30	40	40	30:1	8.22	8.28	8.26
7	30	50	40	20:1	9.13	9.18	9.15
8	30	40	30	20:1	8.81	8.77	8.80
9	40	60	30	30:1	8.68	8.66	8.71
10	30	50	20	40:1	9.16	9.14	9.16
11	30	50	30	30:1	11.12	10.95	10.98
12	30	60	20	30:1	8.51	8.49	8.52
13	30	40	30	40:1	9.15	9.13	9.15
14	40	50	20	30:1	8.17	8.27	8.09
15	30	40	20	30:1	8.69	8.67	8.70
16	40	50	40	30:1	8.41	8.38	8.42
17	30	50	30	30:1	10.96	10.95	10.98
18	40	50	30	40:1	9.16	9.15	9.16
19	30	50	30	30:1	10.58	10.95	10.98
20	20	50	30	20:1	8.48	8.53	8.51
21	30	50	40	40:1	9.44	9.47	9.42
22	30	50	30	30:1	11.25	10.95	10.98
23	20	50	20	30:1	7.76	7.72	7.74
24	20	50	40	30:1	8.34	8.17	8.37
25	30	50	30	30:1	10.83	10.95	10.98
26	30	60	40	30:1	9.38	9.44	9.42
27	40	50	30	20:1	8.47	8.46	8.48
28	30	60	30	40:1	9.53	9.50	9.52
29	30	50	20	20:1	8.96	8.96	8.97

**Table 2:** ANOVA results for regression equation.

Source	Sum of Squares	*df*	Mean Square	F Value	*p* Value	Significance
Model	28.28	14	2.02	83.16	<0.0001	Significant
A	0.4294	1	0.4294	17.68	0.0009	**
B	0.7301	1	0.7301	30.06	<0.0001	**
C	0.2324	1	0.2324	9.57	0.0079	**
D	0.1680	1	0.1680	6.92	0.0198	*
AB	0.0256	1	0.0256	1.05	0.3220	
AC	0.0289	1	0.0289	1.19	0.2938	
AD	0.2070	1	0.2070	8.52	0.0112	*
BC	0.4489	1	0.4489	18.48	0.0007	**
BD	0.0144	1	0.0144	0.5928	0.4542	
CD	0.0030	1	0.0030	0.1245	0.7294	
A^2^	18.60	1	18.60	765.84	<0.0001	**
B^2^	7.99	1	7.99	328.88	<0.0001	**
C^2^	8.12	1	8.12	334.09	<0.0001	**
D^2^	2.68	1	2.68	110.17	<0.0001	**
Residual	0.3401	14	0.0243			
Lack of fit	0.0698	10	0.0070	0.1033	0.9982	Not significant
Pure Error	0.2703	4	0.0676			
Cor total	28.62	28				
R^2^	0.9881		Adj R^2^	0.9762		
C.V. %	1.72					

* Indicates significant difference (*p* < 0.05); ** indicates highly significant difference (*p* < 0.01).

**Table 3:** Comparison of optimal extraction conditions and predictive metrics between RSM and GA–BP.

		RSM	GA–BP
Optimum conditions	Water content (%)	35.88	35.46
	Ultrasound temperature (°C)	51.37	51.68
	Ultrasound time (min)	30.63	30.64
	Liquid–solid ratio (mL/g)	31.91:1	31.53:1
BFP yield (%)	Predicted value	10.52	10.55
	Actual value	10.56 ± 0.17	10.58 ± 0.21
Model evaluation metrics	R^2^	0.9881	0.9919
	RMSE	0.0870	0.0722
	MAE	0.0621	0.0428

**Table 4:** Extraction rate constant *k* at various temperatures and liquid–solid ratios.

Liquid–Solid Ratio (mL/g)	Temperature/K	Fitted Linear Equation	R^2^	k/min^−1^
10:1	303	ln[C_∞_/(C_∞_ − C_t_)] = 0.0643t + 0.4548	0.9746	0.0643
	313	ln[C_∞_/(C_∞_ − C_t_)] = 0.0724t + 0.4054	0.9909	0.0724
	323	ln[C_∞_/(C_∞_ − C_t_)] = 0.0769t + 0.3637	0.9900	0.0769
20:1	303	ln[C_∞_/(C_∞_ − C_t_)] = 0.0653t + 0.5262	0.9938	0.0653
	313	ln[C_∞_/(C_∞_ − C_t_)] = 0.0709t + 0.5118	0.9932	0.0709
	323	ln[C_∞_/(C_∞_ − C_t_)] = 0.0787t + 0.4863	0.9970	0.0787
30:1	303	ln[C_∞_/(C_∞_ − C_t_)] = 0.0661t + 0.5135	0.9794	0.0661
	313	ln[C_∞_/(C_∞_ − C_t_)] = 0.0699t + 0.5490	0.9904	0.0699
	323	ln[C_∞_/(C_∞_ − C_t_)] = 0.0778t + 0.4983	0.9954	0.0778

**Table 5:** Exponential decay fitting parameters for relative residual rate (Y) at different temperatures and liquid–solid ratios.

Liquid–Solid Ratio (mL/g)	Temperature/K	Fitted Exponential Equation	R^2^
10:1	303	Y = 0.6346·e^−0.0643t^	0.9746
	313	Y = 0.6667·e^−0.0724t^	0.9909
	323	Y = 0.6951·e^−0.0769t^	0.9900
20:1	303	Y = 0.5908·e^−0.0653t^	0.9938
	313	Y = 0.5994·e^−0.0709t^	0.9932
	323	Y = 0.6149·e^−0.0787t^	0.9970
30:1	303	Y = 0.5984·e^−0.0661t^	0.9794
	313	Y = 0.5775·e^−0.0699t^	0.9904
	323	Y = 0.6076·e^−0.0778t^	0.9954

**Table 6:** Relationship between –ln *k* and 1/T at different liquid–solid ratios.

Liquid–Solid Ratio (mL/g)	Fitted Linear Equation	R^2^	E_a_ (kJ/mol)
10:1	−ln k = 0.8772/T + 0.1597	0.9712	7.29
20:1	−ln k = 0.9147/T + 0.2853	0.9922	7.61
30:1	−ln k = 0.7890/T + 0.1212	0.9601	6.56

**Table 7:** Physicochemical properties of fresh and recovered ChCl–U DES.

Sample	pH	Viscosity (mPa·s)	Water Content (*w*/*w*%)
Fresh	9.8	10.4	35
Recovered	8.7	12.8	35

**Table 8:** Chemical composition and monosaccharide profiles of HWE–BFPs and UAE–DES–BFPs.

Name	Total Sugar (%)	Protein (%)	Uronic Acid (%)	Monosaccharide Composition (Molar Percentage%)						
				Man	Glc	Rha	GalA	Gal	Xyl	Ara
UAE–DES	78.26 ± 0.29	2.12 ± 0.13	6.24 ± 0.26	7.82	17.35	6.50	4.98	31.90	9.22	22.23
HWE	80.32 ± 0.24	2.58 ± 0.15	2.76 ± 0.18	14.53	34.58	4.83	3.14	21.33	7.60	14.01

**Table 9:** Molecular weight distributions and mass proportions of polysaccharide fractions from UAE–DES–BFPs and HWE–BFPs.

Name	Peaks	Peak Area Percentage (%)	Molecular Weight (kDa)
UAE–DES	1	74.7	171.75
	2	25.3	40.73
HWE	1	78.8	192.71
	2	21.2	48.41

## Data Availability

Data are contained within the article.

## Supplementary Materials

The following supporting information can be downloaded at: https://www.mdpi.com/article/10.3390/molecules31214008/s1, Table S1. Compositions of various DESs. Table S2. Factor levels for Box-Behnken response surface design.
