# Supplementary material for: Ultrasound-Assisted Deep Eutectic Solvent Extraction of Polysaccharides from Baeckea frutescens L.: Hybrid RSM and GA–BP Optimization, Structural Characterization, and Anti-Complementary Activity
Source: Molecules. 2026 Nov 6;31(21):4008. doi: 10.3390/molecules31214008 (PMC13765370; doi:10.3390/molecules31214008)
Supplement: Supplementary file 1 [file molecules-31-04008-s001.zip › molecules-4646682-supplementary.pdf]

Supplementary Material

Ultrasound-assisted deep eutectic solvent extraction of *Baeckea frutescens* L. polysaccharides: Hybrid RSM and GA-BP optimization, structural characterization, and anti-complementary activity

Ming Bu [1], Haijun Wang [1], Yukun Ma [2], Tianci Xu [1], Lu Wang 1, Lina Guo [1] and Jiaming Wang [1,*]

1 College of Pharmacy, Qiqihar Medical University, Qiqihar 161006, China

2 Research Institute of Medicine & Pharmacy, Qiqihar Medical University, Qiqihar 161006, China

* Correspondence: wangjiaming@qmu.edu.cn; Tel.: +86-0452-2663881

Contents

3. Materials and methods

3.1. Materials and reagents

Table list

Table S1. Compositions of various DESs.

Table S2. Factor levels for Box-Behnken response surface design.

3. Materials and methods

3.1. Materials and reagents

Normal human serum pool (NHSP) was collected from a group of healthy volunteer donors. Sheep and rabbit erythrocytes were harvested, with Alsever's solution applied as the preservation medium. Rabbit haemolysin was supplied by Sbjbio (Nanjing, China). Human complement-depleted sera were purchased from Complement Technology (Texas, USA). Gelatine veronal buffer and its variants (GVB^{++}, GVB-Mg^{2+}/EGTA) were prepared according to standard laboratory protocols. All chemical substances including choline chloride, citric acid, malic acid, lactic acid and glycerol were of analytical purity grade. Sinopharm Chemical Reagent Co., Ltd. (Shanghai, China) supplied these chemical reagents. Monosaccharide standards were obtained from Sigma-Aldrich (St. Louis, MO, USA). 3-Phenyl-5-methylpyrazolone (PMP) was provided by Aladdin Biochemical Technology Co., Ltd. (Shanghai, China). Bovine serum albumin (BSA) and Coomassie Brilliant Blue G-250 were acquired from Solarbio Science & Technology Co., Ltd. (Beijing, China). Horseradish peroxidase (HRP)-labelled goat anti-human C3c and C4c antibodies were obtained from Complement Technology (Tyler, TX, USA). The TMB chromogenic substrate utilized in the immunoassay was sourced from Beyotime Institute of Biotechnology (Shanghai, China).

Table S1. Compositions of various DESs.

Number	HBA	HBD	Molar Ratio (HBA:HBD)	Reference
DES-1		Ethylene glycol		[Li et al., 2024]
DES-2		Glycerol	1:2	[Li et al., 2024]
DES-3		Urea		[Li et al., 2024]
DES-4		Lactic acid	1:1	[Li et al., 2024]
DES-5		Malic acid		[Yang et al., 2023]
DES-6	Choline chloride	Citric acid		[Yang et al., 2023]
DES-7		Oxalic Acid		[Yang et al., 2023]
DES-8		1, 3-Butanediol	1:2	[Mahmood et al., 2019]
DES-9		1, 4-Butanediol		[Li et al., 2024]
DES-10		1, 2-Propanediol		[Li et al., 2024]
DES-11		1, 3-Propanediol		[Mahmood et al., 2019]

Table S2. Factor levels for Box-Behnken response surface design.

Level	Factors			
	**(A) water content/%**	**(B) ultrasound temperature/°C**	**(C) ultrasound time/min**	**(D) liquid-to-solid ratio/mL/g**
–1	20	40	20	20:1
0	30	50	30	30:1
1	40	60	40	40:1

References

50. Li, S.; Wang, G.; Zhao, J.; Ou, P.; Yao, Q.; Wang, W. Ultrasound-assisted extraction of phenolic compounds from Celtuce (*Lactuca sativa* var. *augustana*) leaves using natural deep eutectic solvents (NADES): process optimization and extraction mechanism research. *Molecules* **2024**, 29(10), 2385.

51. Yang, D.; Qiu, W.J.; Xu, Y.; Hu, Z.L.; Wang, L.Y. Optimisation and modelling of ultrasonic-assisted extraction of canthaxanthin from *Chromochloris zofingiensis* using eutectic solvents. *Ind. Crops Prod.* **2023**, 202, 117002.

52. Mahmood, W.M.A.W.; Lorwirachsutee, A.; Theodoropoulos, C.; Gonzalez-Miquel, M. Polyol-based deep eutectic solvents for extraction of natural polyphenolic antioxidants from *Chlorella vulgaris*. *ACS Sustain. Chem. Eng.* **2019**, 7, 5018–5026.
